# Supplementary material for: Sympathetic Overactivation Drives Colonic Eosinophil Infiltration Linked to Visceral Hypersensitivity in Irritable Bowel Syndrome
Source: Cell Mol Gastroenterol Hepatol. 2025 Oct 9;20(3):101658. doi: 10.1016/j.jcmgh.2025.101658 (PMC12719208; doi:10.1016/j.jcmgh.2025.101658)
Supplement: Supplementary Data [file mmc1.pdf]

Supplemental Table 1: Primary antibodies used for immunohistochemical staining

Antigen	Host	Manufacture	Cat. number	Dilution
Cre Recombinase	Mouse	Sigma-Aldrich	MAB3120	1:500
RFP	Rabbit	MBL	PM005	1:1000
eotaxin-1	Mouse	Santa Cruz	Sc-373767	1:50
MBP	Mouse	Bio-Rad	MCA5751	1:500
tryptase	Mouse	Abcam	ab2378	1:500
MPO	Rabbit	Abcam	Ab9535	1:1000
Iba1	Rabbit	Wako	019-1074	1:1000
CD68	Rabbit	Abcam	Ab2512	1:1000
CD31	Mouse	Millipore	MAB1393	1:1000
vimentin	Rabbit	Abcam	Ab92547	1:5000

Supplemental Table 2: Secondary antibodies used for immunohistochemical staining

Antibody	Manufacture	Cat. number	Dilution
Biotinylated-horse anti- mouse IgG	Vector	BA2001	1:200
Biotinylated-goat anti-rabbit IgG	Vector	BA1000	1:200
Donkey anti-rabbit Alexa Fluor 594	Molecular Probes	A32754	1:1000
Goat anti-rabbit Alexa Fluor 594	Molecular Probes	A11037	1:1000
Goat anti-mouse Alexa Fluor 488	Molecular Probes	A11029	1:1000
Donkey anti-goat Alexa Fluor 488	Molecular Probes	A11055	1:1000

Supplemental Table 3: Sequences of primers used in PCR

Gene	Accession No.	Type	Sequence (5′–3′)
hccl11	NM_002986.3	sense	AAGCTCACACCTTCAGCCTC
		antisense	CACTCAGGCTCTGGTTTGGT
rccl11	NM_019205.2	sense	CAGCTCTCCACAGCACTTCT
		antisense	GGGTGCCGATATTCTCCCAT
rccl24	NM_001013045.1	sense	CTTGCACCCCAGCTTTGAAC
		antisense	GGTGCTATTGCCTCGGAGTT
rccl26	XM_006249188.4	sense	GGTTCTTGAGCGTCCACACA
		antisense	TGGCTGGACACAGTATTGCT
rIL5	NM_021834.1	sense	GATGCTTCTGTGCTTGAGCG
		antisense	TCTTCGCCACACTGCTCTTT
rIL33	NM_001014166.1	sense	CTGCACAATCAGGAGACGGT
		antisense	CCCAGAAGGCACAGACCTTT
rPrg3	XM_031373412.1	sense	GCAGGGGAGAGAGTTGGTTC
		antisense	GACGCCTCCAATCCAGACAA
rTpsab1	NM_019322.2	sense	TGACTTCTACATCGCCCAGG
		antisense	Prg-GGCAGGAGTCATGTCCTTCA
rGAPDH	M17701	sense	CCAGGGCTGCCTTCTCTTGT
		antisense	CCAGCCTTCTCCATGGTGGT
mAdrb1	NM_007419.3	sense	TCTGGTCATGGGATTGCTGG
		antisense	CCTGTTGGTGACGAAATCGC
mAdrb2	NM_007420.3	sense	GAACGACAGCGACTTCTTGC

		antisense	GATCCACTGCAATCACGCAC
mAdrb3	NM_013462.3	sense	GAGTGAGTCCCCTGGAACCT
		antisense	AGTGAGGAGACAGGGATGAAA
mAdra1a	NM_001271760.1	sense	CAACCCGAGCTGCAAAGTTC
		antisense	GGAAACGTGAGCCTGAGGAA
mAdra1b	XM_011248675.4	sense	ATTGAAAGCAGACCCTCCTCG
		antisense	GCAGGTGCTGATGTGTTGTG
mAdra1d	NM_013460.5	sense	GCCACTCGCTCAAGTATCCA
		antisense	GACGATGGCTAGGGTCTTGG
mAdra2a	NM_007417.5	sense	CGCTGGACCTAGAGGAGAGT
		antisense	TTCAGCGAGCTGTTGCAGTA
mAdra2b	NM_009633.4	sense	CCAACAGTAGCGGAGCTAGG
		antisense	TGTCCAGACTGATGGCACAC
mAdra2c	NM_007418.3	sense	TCACCGTGGTAGGCAATGTG
		antisense	GCGGTAGAACGAGACGAGAG
mCD45	NM_001268286.1	sense	CCAGTGATGCTACCACAACGA
		antisense	GCACGAAGGTTGTCCAACTG
mCD31	NM_001032378.2	sense	GTGAATGACACCCAAGCGTT
		antisense	GAGCCTTCCGTTCTCTTGGT
mCD34	NM_001111059.2	sense	ACAGTACCTCACAACCCTGC
		antisense	GTCCAGGGCAAGTGCTACAT
m Pdgfra	NR_144636.1	sense	CACTTTGACCGTCCCCAAGG
		antisense	CATCCCGACCACACAAGAACA

mGAPDH	NM_001411843.1	sense	GTGTGAACGGGTGAGTTCCA
		antisense	GTCTCGCTCCTGGAAGATGG
hAdrb1	NM_000684.3	sense	ACTCGAAGCCCACAATCCTC
		antisense	TCTGGCTGGTAGTGTGTTCC
hAdrb2	NM_000024.6	sense	CTCTCATCGTCCTGGCCATC
		antisense	GAATGATCACCCGGGCCTTA
hAdrb3	NM_000025.3	sense	GCCAATTCTGCCTTCAACCC
		antisense	TCGTCAGGTTCTGGAGGGTA
hAdra1a	NM_001322502.1	sense	AATGCTTCCGACAGCTCCAA
		antisense	GATGATGCAGAGGCCCATGA
hAdra1b	NM_000679.4	sense	CTGTTGAGCTTCACCGTCCT
		antisense	ATCCTCAGGGTCAGCTCCTT
hAdra1d	NM_000678.4	sense	ACTCACTCAAGTACCCAGCC
		antisense	CTCACGGGAGAACTTGAGCA
hAdra2a	NM_000681.4	sense	GGCTACTGGTACTTCGGCAA
		antisense	CTGGTAGATGCGCACGTAGA
hAdra2b	NM_000682.7	sense	TCTGGATCGGCTACTGCAAC
		antisense	AAGGGAAGCCCAGACATTGG
hAdra2c	NM_000683.4	sense	GCCCGCTCTTCAAGTTCTTC
		antisense	CCTTGCTTGCCCATTTAGGG
hGAPDH	NM_001357943.2	sense	ACACCATGGGGAAGGTGAAG
		antisense	TGGTTCACACCCATGACGAA
