# Supplementary material for: Sympathetic Overactivation Drives Colonic Eosinophil Infiltration Linked to Visceral Hypersensitivity in Irritable Bowel Syndrome
Source: Cell Mol Gastroenterol Hepatol. 2025 Oct 9;20(3):101658. doi: 10.1016/j.jcmgh.2025.101658 (PMC12719208; doi:10.1016/j.jcmgh.2025.101658)
Supplement: Extended [file mmc2.pdf]

cmgh
CELLULAR AND MOLECULAR GASTROENTEROLOGY AND HEPATOLOGY

ORIGINAL RESEARCH

Sympathetic Overactivation Drives Colonic Eosinophil Infiltration Linked to Visceral Hypersensitivity in Irritable Bowel Syndrome

Shaoqi Duan,[1] Hirosato Kanda,[2,3] Feng Zhu,[1] Masamichi Okubo,[1,2] Taro Koike,[4] Yoshiya Ohno,[5] Toshiyuki Tanaka,[5] Yukiko Harima,[6] Kazunari Miyamichi,[6] Hirokazu Fukui,[7] Shinichiro Shinzaki,[7] Yilong Cui,[1] Koichi Noguchi,[1,2] and Yi Dai[1,2]

[1]Department of Anatomy and Neuroscience, School of Medicine, Hyogo Medical University, Nishinomiya, Japan; [2]Laboratory of Basic Pain Research, School of Pharmacy, Hyogo Medical University, Kobe, Japan; [3]Laboratory of Anatomy, School of Pharmacy, Hyogo Medical University, Kobe, Japan; [4]Department of Anatomy, School of Medicine, Kansai Medical University. Hirakata, Japan; [5]Laboratory of Immunobiology, School of Pharmacy, Hyogo Medical University, Kobe, Japan; [6]Laboratory for Comparative Connectomics, RIKEN Center for Biosystems Dynamics Research, Kobe, Japan; and [7]Department of Gastroenterology, School of Medicine, Hyogo Medical University, Nishinomiya, Japan

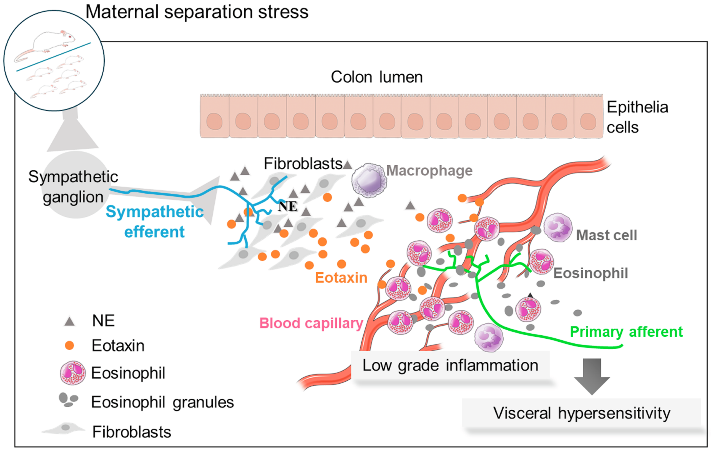

SUMMARY

This study, employing animal models, chemogenomic modulation, and clinical biopsies, demonstrates that sympathetic overactivation promotes eosinophil infiltration into the colon via fibroblast-mediated eotaxin-1 signaling, contributing to visceral hypersensitivity in inflammatory bowel syndrome.

WHAT YOU NEED TO KNOW

Background: A subgroup of patients with inflammatory bowel syndrome (IBS) shows gastrointestinal immune alterations, yet the role of sympathetic nervous system-induced immune dysregulation in this process remains unclear.

Impact: Using animal models, this study provides novel insights into the critical role of sympathetic nervous system overactivation in driving mesenchymal cell-mediated colonic eosinophil infiltration, ultimately contributing to the development of visceral hypersensitivity in IBS.

Future directions: Future studies should explore whether targeting fibroblast-derived eotaxin-1 or inhibiting eosinophil infiltration may represent promising therapeutic strategies to alleviate visceral pain in patients with IBS.

BACKGROUND & AIMS: Mucosal immune alteration is a characteristic clinical manifestation of irritable bowel syndrome (IBS), and its symptoms are often triggered by psychological stress. The present study aimed to investigate the impact of early life stress-associated dysfunction of the sympathetic nervous system (SNS) on mucosal immune changes in the gastrointestinal tract (GI) and its contribution to visceral hypersensitivity of IBS.

METHODS: We utilized a traditional animal model of IBS with maternal separation (MS) and evaluated colorectal hypersensitivity, immune alteration, and SNS activity in adult rats with

MS. We conducted a series of experiments to manipulate peripheral SNS activity pharmacologically and chemogenetically to explore the interaction between SNS activity and GI events.

RESULTS: The MS-induced IBS model exhibited visceral hypersensitivity and eosinophilic infiltration in the colonic mucosa, along with SNS overactivation. Degeneration of the SNS using 6-OHDA neurotoxin decreased eosinophil infiltration and visceral hypersensitivity in the MS model. Notably, specific chemogenetic activation of the peripheral SNS induced eosinophil infiltration in the intestinal mucosa through the noradrenergic signaling-mediated release of eotaxin-1 from mesenchymal cells.

CONCLUSIONS: This study highlights the critical role of SNS overactivation in eotaxin-1-driven eosinophil infiltration in the colon, leading to the development of visceral hypersensitivity in IBS. The results provide important insights into the mechanistic links among increased sympathetic activity, mucosal immune alteration, and visceral hypersensitivity in individuals with IBS, suggesting potential therapeutic approaches. *(Cell Mol Gastroenterol Hepatol 2026;20:101658; https://doi.org/10.1016/j.jcmgh.2025.101658)*

Keywords: Eosinophil; Eotaxin-1; Irritable Bowel Syndrome; Sympathetic Nervous System.

This article has an accompanying editorial.

Irritable bowel syndrome (IBS) is a prevalent functional gastrointestinal disorder (FGID) characterized by intermittent or persistent abdominal pain, diarrhea, and constipation without apparent organ abnormalities.[1] These recurring symptoms often impact the quality of life and are closely associated with mental stress. Although IBS affects approximately 15% of the global population,[2] effective clinical treatment remains challenging owing to a limited understanding of its underlying pathophysiological mechanism.

The pathogenesis of IBS involves a complex interplay of genetic, environmental, and immunological factors.[3] Various pathological mechanisms, including visceral hypersensitivity, altered intestinal motility, immune dysfunction, and dysbiosis, are implicated in IBS.[4] Notably, some patients with IBS exhibit microinflammation (low-grade inflammation) in the gastrointestinal (GI) tract mucosa,[5] which challenges the conventional view that FGIDs lack organic abnormalities. Clinical evidence indicates that a considerable subgroup of individuals with IBS experiences microinflammation in the colonic mucosa, characterized by infiltration and activation of immune cells within the GI mucosa.[6,7] Mast cells have garnered attention owing to their contribution to visceral hypersensitivity.[8–10] Additionally, higher numbers of eosinophils, lymphocytes, and other plasma cells have been reported in the intestinal mucosae of patients with IBS than in those of healthy individuals.[4,5,11,12] These reported variations in the types of immune cells contributing to microinflammation may be attributed to the geographic differences in diet, genetic backgrounds, patient phenotypes, or biopsy sites.[13] Despite numerous reports of increased immune cell numbers in the mucosa of patients with IBS, the underlying mechanisms inducing alterations in the intestinal immune system remain unknown.

Psychosocial factors (particularly early life adverse events and traumatic stress) are strongly implicated in the pathophysiology of IBS. Specifically, individuals exposed to stress in early life are more likely to develop IBS during adulthood.[14,15] In a subset of patients with IBS who have experienced early life stress, the sympathetic nervous system (SNS) becomes more sensitive to stress, leading to sympathetic overactivation.[16–19] The mechanisms underlying the influence of psychosocial events on IBS have been investigated, with particular focus on the role of the hypothalamic-pituitary-adrenal (HPA) axis dysregulation, a neuroendocrine pathway.[20–22] However, the contribution of the peripheral SNS remains unknown. Emerging evidence indicates an interaction between the SNS and the immune system.[23] The SNS uses norepinephrine (NE) as its primary neurotransmitter to regulate communication between sympathetic neurons and immune cells. Generally, SNS activation suppresses immune responses.[24,25] However, in certain cases, NE signaling induces immune cell accumulation in local tissues, subsequently enhancing immune responses.[26,27] Therefore, the SNS can exert both activating and inhibitory effects on immunity.[23] The occurrence of both immune cell alterations and sympathetic perturbation in patients with IBS suggests a potential causal relationship between an overactive SNS and immune alteration, contributing to IBS pathogenesis.

We have previously reported that maternal separation (MS) stress induces eosinophil-associated microinflammation in the gastroduodenal mucosa, which is linked with increased gastric hypersensitivity to distention.[28] Mucosal eosinophils are key neuroimmune players

Abbreviations used in this paper: **AAV, adeno-associated virus; ANOVA, analysis of variance; AP, alkaline phosphatase; AR, adrenergic receptor; AUC, area under the curve; BSA, bovine serum albumin; Cartpt, cocaine- and amphetamine-regulated transcript protein; CG-SMG, celiac-superior mesenteric ganglion; CNO, clozapine N-oxide; CNS, central nervous system; CRD, colorectal distension; DAB, 3, 3′-diaminobenzidine; DAPI, 4',6-diamidino-2-phenylindole; DBH, dopamine beta hydroxylase; DEX, dexamethasone; DSMO, dimethylsulphoxide; ELISA, enzyme-linked immunosorbent assay; EMG, electromyography; ENS, enteric nervous system; EPO, eosinophil peroxidase; FACS, fluorescence-activated cell sorting; FBS, fetal bovine serum; FGID, functional gastrointestinal disorder; GI, gastrointestinal; H&E, hematoxylin and eosin; HBSS, Hank's balanced salt solution; HPA, hypothalamic-pituitary-adrenal; Iba-1, ionized calcium-binding adapter molecule 1; IBS, irritable bowel syndrome; IHC, immunohistochemistry; IL, interleukin; ir, immunoreactive; MBP, major basic protein; MPO, myeloperoxidase; MS, maternal separation; NE, norepinephrine; OPD, o-phenylenediamine; PBS, phosphate buffered saline; PVN, paraventricular nucleus; RT-PCR, reverse-transcription polymerase chain reaction; SCG, superior cervical ganglion; SD, standard deviation; SE, standard error; SNS, sympathetic nervous system; Veh, vehicle; VMR, visceromotor response.**

Most current article

© 2025 The Authors. Published by Elsevier Inc. on behalf of the AGA Institute. This is an open access article under the CC BY license (http://creativecommons.org/licenses/by/4.0/).
2352-345X
https://doi.org/10.1016/j.jcmgh.2025.101658

in regulating GI function and are implicated in brain-gut interaction disorders.[29] Herein, we used the MS model to mimic the eosinophil infiltration observed in patients with IBS and examined the influence of SNS overactivity on immune alteration of gastrointestinal tract. Specifically, we introduced 2 chemogenomic approaches to manipulate SNS activity, which enabled the manipulation of peripheral sympathetic activity without the confounding effects of glucocorticoids.

Results

MS Stress-induced IBS Model Rats Exhibit Eosinophil Infiltration and SNS Overactivation

We used a traditional MS stress-induced IBS model to induce colonic immune alterations and visceral hypersensitivity. Adult MS rats exhibited visceral hypersensitivity, evidenced by increased visceromotor response (VMR) at 40 mmHg and 60 mmHg compared with control rats (Figure 1*A, B*). The threshold of the colorectal distention (CRD) response exhibited no significant differences between MS and control groups (Figure 1*C*), indicating that MS stress induced visceral hyperalgesia rather than allodynia in adulthood. These data confirm that MS stress induces visceral hypersensitivity to CRD in adulthood, a characteristic symptom of IBS.

Immune alteration has been reported in patients with FGIDs, relating to clinical symptom severity.[4] We observed a significantly higher number of major basic protein (MBP)-immunoreactive (-ir) cells (marker for eosinophil) in the colonic tissue of the MS group than in the controls (Figure 1*D, E*). Furthermore, elevated eosinophil peroxidase (EPO) activity was observed in the colon of the MS group, reflecting an increased activation status of eosinophils (Figure 1*F*). Although there was a trend toward an increase in the number of tryptase-ir cells ($P = .138$, a marker for mast cells), the difference between groups was not statistically significant. The number of myeloperoxidase (MPO)-ir (marker for neutrophil) and ionized calcium-binding adapter molecule 1(Iba-1)-ir cells (marker for macrophage) did not significantly differ between the control and MS groups (Figure 1*D, E*). The relative mRNA expression of MBP (*Prg3*) rather than that of tryptase (*Tpsab1*) was increased in the colon of the MS model, suggesting a predominance of eosinophil-related activity (Figure 1*G*). Notably, no apparent morphological alterations were observed in the colonic mucosal architecture of the MS group (Figure 1*H*). These findings demonstrate that early-life stress induces eosinophil infiltration and activation in the colon.

Neuroimmune interactions are important in the immune response.[23] Given the common feature of increased SNS activity in IBS,[6,7] we quantified SNS activity in the MS group by monitoring NE serum concentration, which was significantly higher than that in the control group (Figure 1*I*). These data indicated that MS rats exhibited an overactive SNS. To investigate whether the overactive sympathetic signal contributes to eosinophil infiltration in the MS model, we blocked sympathetic activity using 6-OHDA (a neurotoxic compound selective for dopaminergic and noradrenergic neurones). Intraperitoneal injection of 6-OHDA (7 days after treatment) significantly suppressed eosinophil infiltration in the MS group (Figure 1*J*). Additionally, treatment with the alpha-adrenergic receptor (AR) antagonist (phentolamine) for 7 continuous days significantly suppressed eosinophil infiltration compared with that in the vehicle (Veh)-treated MS group. Moreover, treatment with phentolamine and propranolol did not show significant eosinophil infiltration in the MS group (Figure 1*K*). These data suggest that sympathetic signaling plays a role in mucosal eosinophil migration to the colon in the MS rats.

Genetic Activation of Peripheral SNS Induces Eosinophil Infiltration in the Colon of Dopamine Beta Hydroxylase-cre Mice

To further investigate the regulatory role of sympathetic activity in eosinophil migration, we employed a genetic manipulation technique to control peripheral sympathetic signals without affecting adrenal corticoid function. We utilized adeno-associated virus (AAV) PHP.S-hSyn-DIO-hM3D-mCherry[30] to modulate peripheral SNS activity in dopamine beta hydroxylase (DBH)-Cre mice (Figure 2*A*). We quantified the infection rate in the superior cervical ganglion (SCG), where AAV PHP.S-hSyn-DIO-hM3D-mCherry infected 24.8 ± 1.14% of neurones in a cre-dependent manner, without any detectable infection in the central nervous system (CNS) and somatosensory system (Figure 2*B, C, and D*). This strategy enabled the targeted activation of DBH-Cre positive cells to modulate peripheral SNS activity, evidenced by a significant reduction in colonic fecal pellet output following clozapine N-oxide (CNO) administration (Figure 2*E*). Additionally, sustained CNO stimulation over 5 days resulted in elevated serum NE levels (Figure 2*F*), further supporting enhanced SNS activity. To study the immune alterations in the colon with chemogenetic modulation of peripheral sympathetic activity, we initially investigated the distribution of eosinophils in colorectal tissue via immunostaining. A significant increase in MBP-ir cell count was observed in the lamina propria of the CNO group compared with the Veh group (Figure 2*G, H*), exhibiting a pattern similar to that observed in the MS model rats.

To confirm the immune alterations more specifically, we conducted flow cytometry on a single-cell suspension prepared from the colon of DBH-cre mice. We first gated CD45+ cells as immune cells (Figure 3*A*) and then used specific cell surface markers to identify target immune cells. The ratio and absolute number of colonic eosinophils ($CD11b^+$ $SiglecF^+$) were significantly increased in the CNO-treated group of DBH-Cre mice infected with AAV-PHP.S-hSyn-DIO-hM3D-mCherry (Figure 3*B, C*). No significant differences were observed in neutrophils ($CD11b^+$ $Ly6G^+$), basophils ($CD11b^+$ $FceRI^+$), macrophages ($CD11b^+$ $F4/80^+$), or mast cells ($CD117^+$ $FceRI^+$) between the CNO and Veh-treated groups. The ratio of $CD4^+$ T cells decreased after CNO treatment. There were no significant changes in the proportion and number of any immune cells after CNO treatment in naïve mice

(Figure 3*D*). The percentage of basophils (CD11b$^+$ FceRI$^+$) was higher than its basal value under normal conditions according to the fluorescence-activated cell sorting (FACS) data. This may be owing to a limitation of the FceRI$^+$ antibody recognizing other cell types when used in rodents.[31]

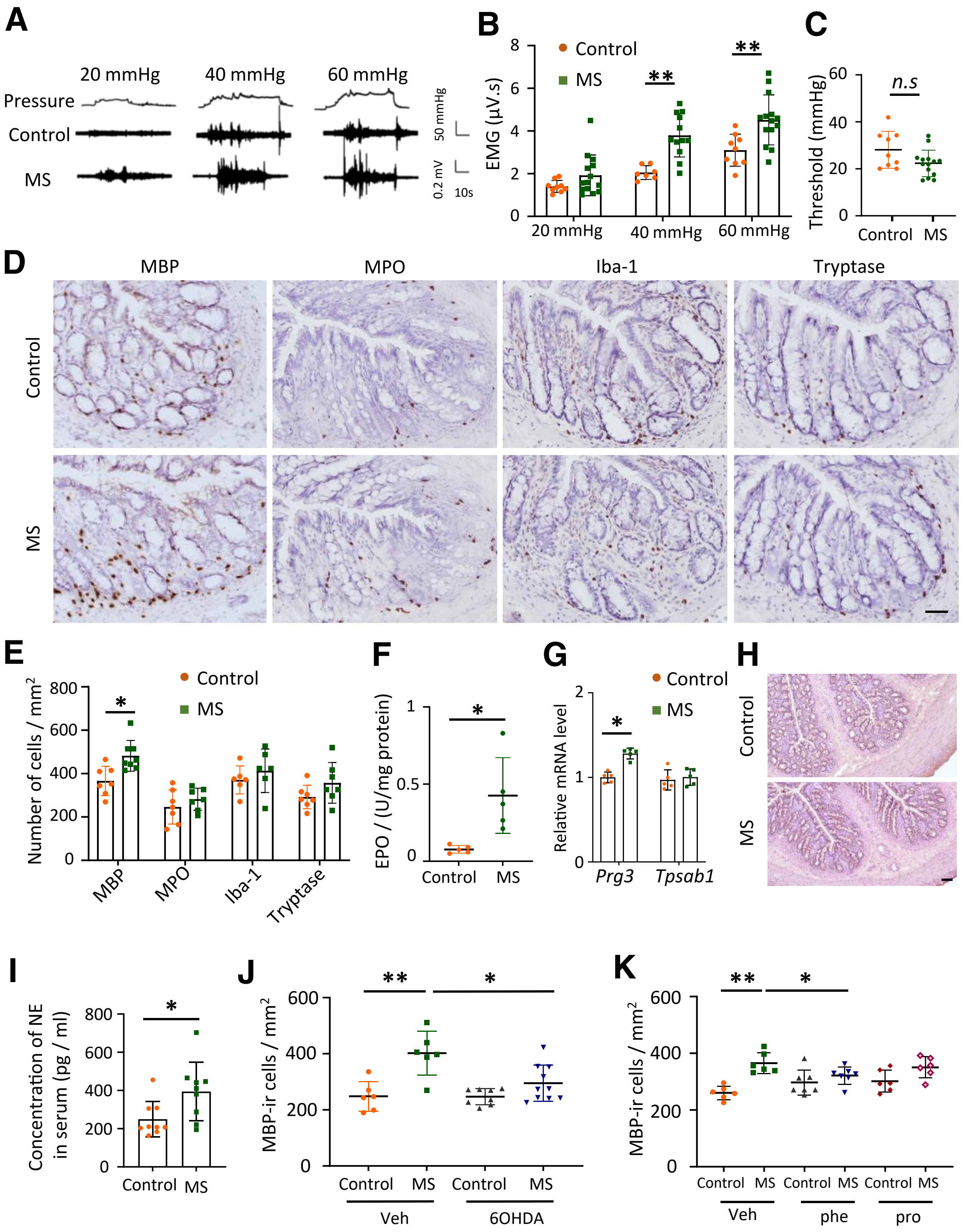

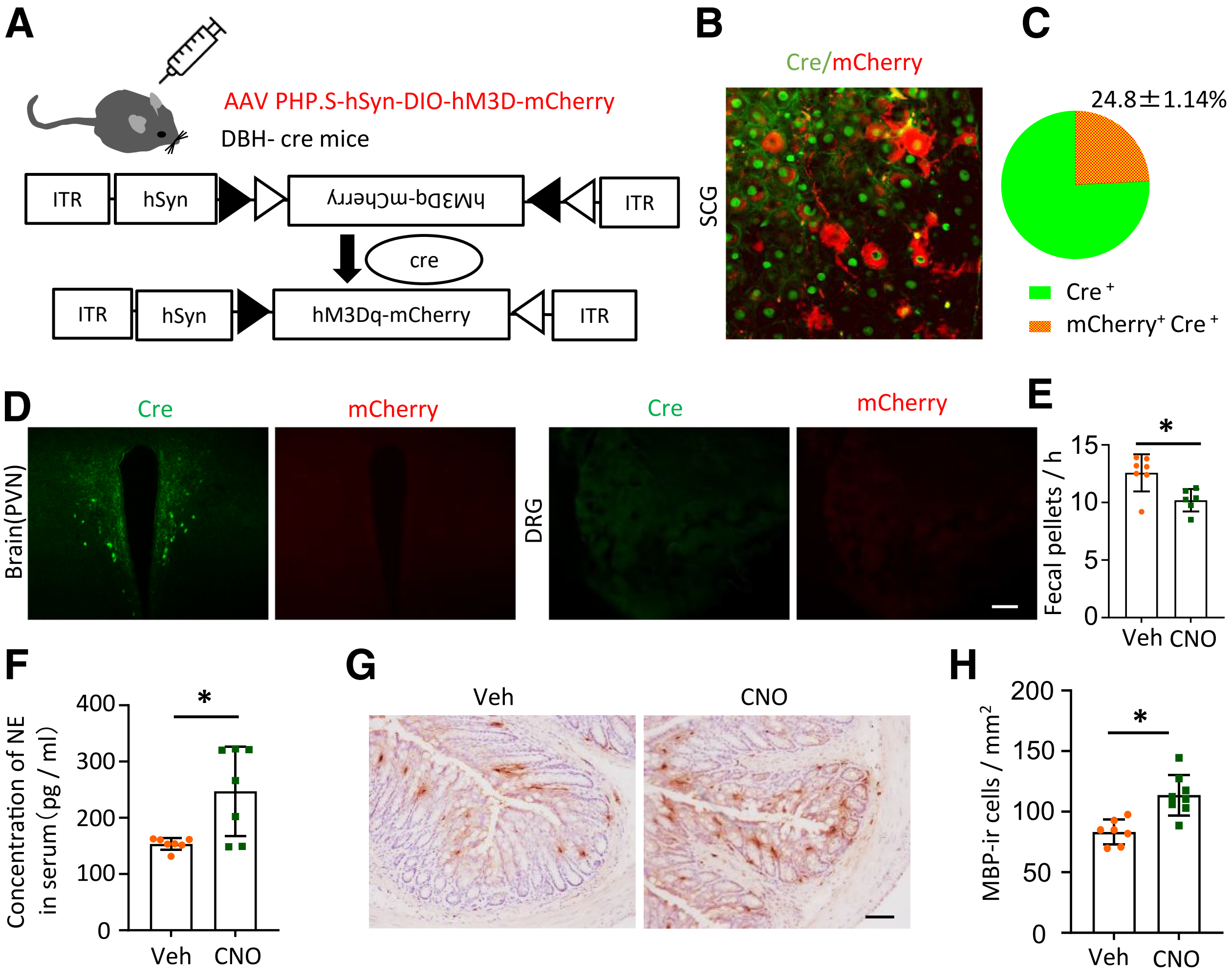

Figure 2. Genetic activation of peripheral DBH-Cre cells induces eosinophil infiltration in the colon of DBH-cre mice. (*A*) Schematic diagram illustrating the AAV-PHP.s-hSyn-DIO-hM3D construct expressing hM3D in DBH-Cre mice to selectively modify peripheral DBH-positive cells. (*B*) Images of SCG neurons coexpressing cre and mCherry. (*C*) The infection rate of AAV-PHP.S was approximately 24.8% ± 1.14%. (*D*) Representative images showing immunofluorescence staining of cre (*green*) and mCherry (*red*) in the brain PVN and DRG after AAV-PHP.S-hSyn-DIO-hM3D-mCherry infection. Note: no mCherry signal was observed in both DRG and PVN. Scale bar, 50 μm. (*E–F*) CNO treatment increases NE concentration in the serum (*E*) and reduces fecal pellet output from the colon (*F*) (n = 7 in each group). (*G*) IHC images showing MBP-ir cells in the colorectal tissues of Veh and CNO-treated DBH-Cre mice. Scale bar: 50 μm. (*H*) Bar graphs showing the number of MBP-ir cells in the colonic tissues of Veh- and CNO-treated DBH-Cre mice. (n = 7 in each group). Data are presented as mean ± SD; non-parametric Mann-Whitney *U* tests were applied to the remaining panels (*E, F, and H*) for statistical analysis. *$P < .05$.

Figure 1. (***See previous page***). **MS stress-induced IBS model rats exhibit eosinophil infiltration and overactivation of the SNS.** (*A*) Colorectal distention pressures of 20, 40, and 60 mmHg were applied to rats in both the control and the MS groups (*top*). EMG traces of VMR to cCRD at different pressures are shown for the control (*middle*) and the MS group (*bottom*). (*B, C*) Bar graphs and dot plots represent the data of VMR to colorectal distention at different pressures (*B*) and the threshold (*C*), respectively (n = 10–14 rats in each group). (*D*) Representative images of colonic tissue stained for MPO$^+$, Iba-1$^+$, tryptase$^+$ and MBP$^+$ cells in the colon of the control and the MS group. *Brown dots* indicate positive cells. Scale bar, 50 μm. (*E*) Bar graphs represent the number of immune cells in the colonic mucosa of control and the MS groups (n = 6–7 in each group). (*F*) EPO activity in the colon of the control and the MS groups (n = 5 rats in each group). (*G*) Bar graphs show the relative mRNA levels of MBP (Prg3) and tryptase (Tpsab1) in the colon of the control and MS rats (n = 5 in each group). (*H*) Representative H&E staining images of colon in control and MS rat. Scale bar, 100 μm. (*I*) Bar graphs show the NE concentration in the serum of the control and the MS groups (n = 9 rats in each group). (*J*) Scatter dot plot data showing the number of MBP-ir cells in the control and MS groups in the presence or absence of 6-OHDA (n = 6–10 rats in each group). (*K*) Scatter dot plot showing the number of MBP-ir cells in the control and MS groups receiving or not receiving administration of AR antagonists (Phe: phentolamine; Pro: propranolol) (n = 6 rats in each group). Data are represented as mean ± SD. Two-way ANOVA with Bonferroni post hoc test was used for (*B, J, K*), and unpaired *t*-test was used for (*C, E, F, G, I*). *$P < .05$, **$P < .01$. n.s, $P > .05$.

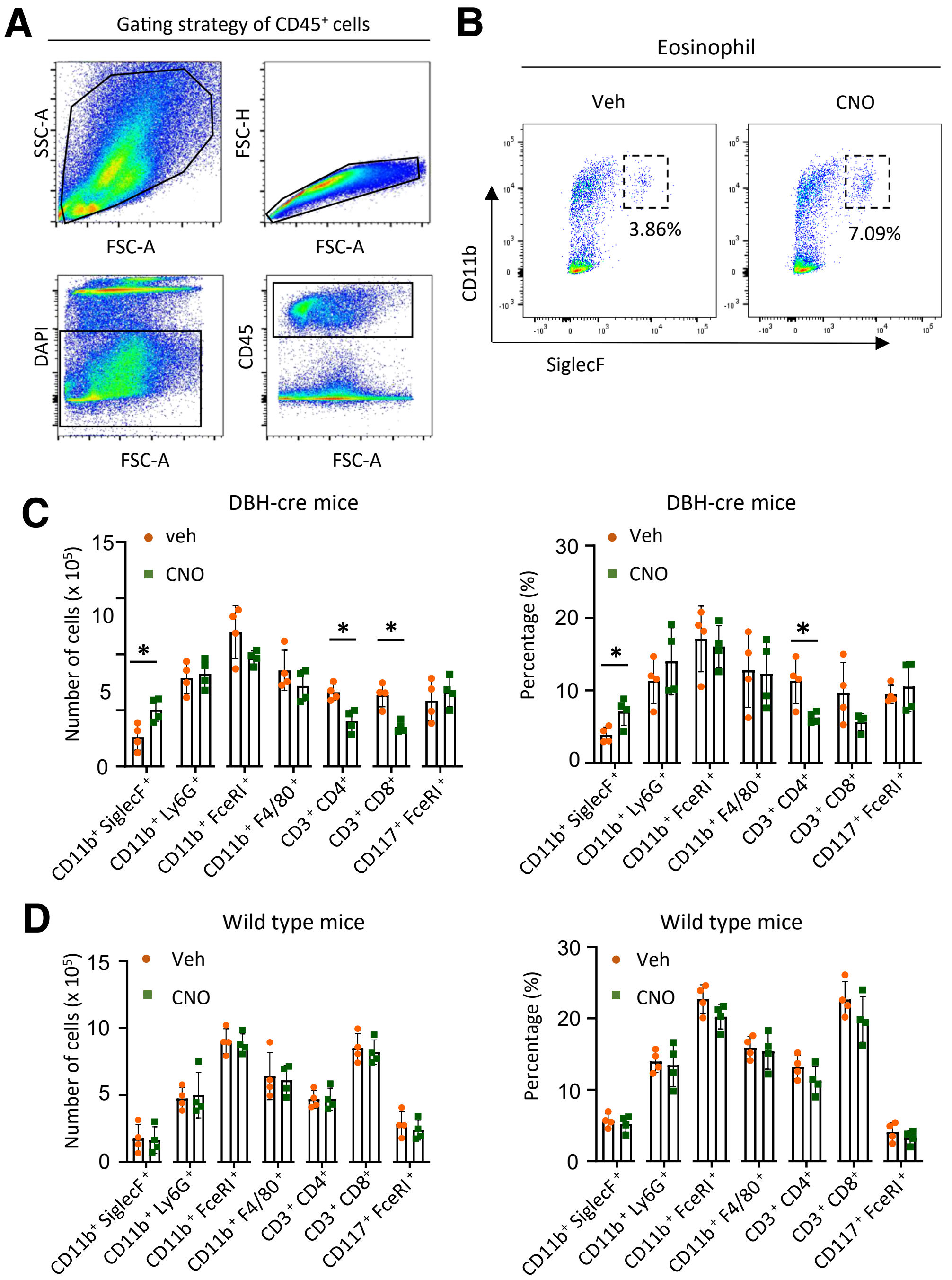

Figure 3. Flow cytometric analysis showing increased eosinophil infiltration in the colon of DBH-cre mice with AAV PHP.S-hSyn-DIO-hM3D-mCherry infection. (*A*) Images showing the gating strategy for $CD45^+$ cells. (*B*) Flow cytometry gated $CD45^+CD11b^+SiglecF^+$ cells as eosinophils in the Veh and CNO-treated DBH-Cre mice. (*C*) Bar graph showing the absolute number and percentage of immune cells in the Veh and CNO-treated DBH-Cre mice. (n = 4 in each group, percentage in CD45+ cells). (*D*) Bar graphs showing the absolute number and percentage of immune cells in the colonic tissue from Veh- or CNO-treated WT mice, as determined by the FACS analysis (n = 4 in each group). Data are presented as mean ± SD; nonparametric Mann-Whitney *U* tests were applied to (*C, D*) for statistical analysis. $^*P < .05$.

Genetic Activation of Peripheral SNS Induces Visceral Pain in DBH-cre Mice

Next, we examined the VMR to CRD at different pressures to assess visceral pain in DBH-cre mice with genetical modulation. The SNS was activated by microinjecting AAV2-hSyn-DIO-hM3D-mCherry into the celiac-superior mesenteric ganglion (CG-SMG) (Figure 4*A, B*) and administering CNO for 5 continuous days. After confirming the elevated eosinophil activation in the CNO-treated group (Figure 4*C*), we found that the VMR to CRD at 60 mmHg was significantly increased in this group compared with the vehicle group (Figure 4*D, E*). Notably, these genetic manipulations did not affect the corticosterone level in the serum of DBH-Cre mice, indicating that the effect on HPA axis was limited (Figure 4*F*). These results provide direct evidence that activation of the SNS contributes to both eosinophil infiltration and visceral pain.

Although chemogenetically activating peripheral SNS using DBH-Cre mice could exclude the effect from the CNS, the peripheral sympathetic nerve-innervating organs, (including the adrenal medulla) would be activated following CNO injection. Therefore, we used cocaine- and amphetamine-regulated transcript protein (Cartpt)-Cre mice with the AAV8-hSyn-DIO-hM3D-mcherry infection to

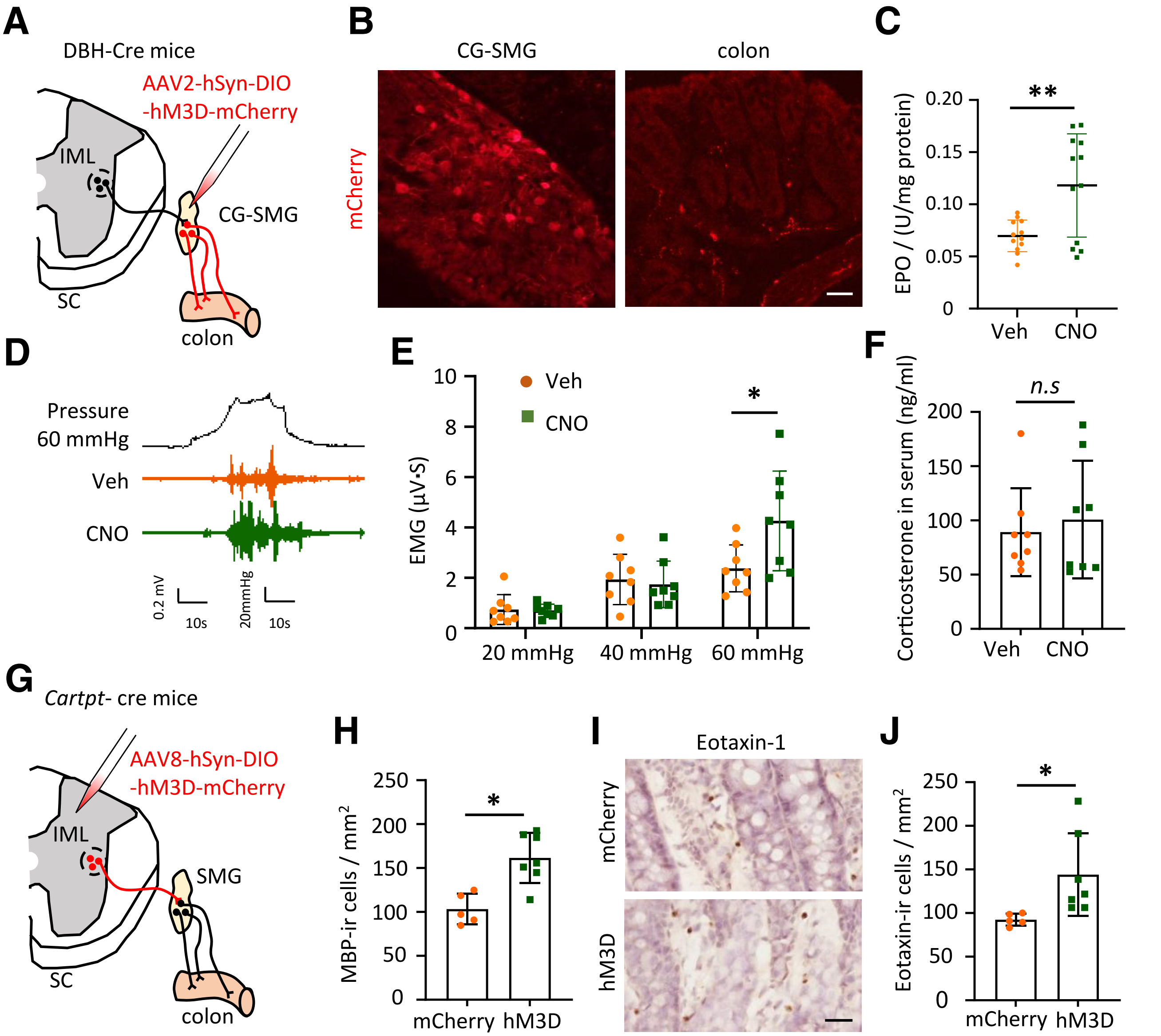

Figure 4. Genetic activation of peripheral DBH-Cre cells induces visceral pain of DBH-cre mice. (*A*) Schematic diagram illustrating the strategy used to activate the SNS. (*B*) Representative images showing AAV-infected, mCherry-immunoreactive neurons in the CG-SMG and distal colon (1–3 cm from the anus) of DBH-Cre mice following CG-SMG microinjection. Scale bar, 20 μm. (*C*) Bar graphs showing the EPO activity of eosinophil from Veh- and CNO-treated DBH-Cre mice (n = 12 in each group). (*D*) Sample trace of VMR to 60 mmHg CRD in Veh- and CNO-treated DBH-Cre mice. (*E*) Bar graph showing the VMR response to CRD at 20, 40, and 60 mmHg in Veh- and CNO-treated DBH-Cre mice (n = 8 in each group). (*F*) Bar graph showing the corticosterone levels in the serum of DBH-Cre mice infected with AAV2-hSyn-DIO-hM3D-mCherry following 5 days of treatment with CNO or saline. (*G*) Schematic diagram illustrating the strategy used to specifically modulate the SNS innervating the colon. (*H*) Bar graphs showing MBP-ir cell counts in the mCherry and hM3D groups of Cartpt-Cre mice following CNO treatment. (*I*) Representative images showing IHC staining of eotaxin-1 expression in colonic tissues of the mCherry and the hM3D-infected Cartpt-Cre mice. (*J*) The bar graph shows the number of eotaxin-1-ir cells in each group. Data are presented as mean ± SD; Two-way ANOVA with Bonferroni post hoc test was used for (*E*), whereas nonparametric Mann-Whitney *U* tests were applied to the remaining panels (*C, F, H and J*) for statistical analysis. *$P < .05$, **$P < .01$.

selectively control the targeted preganglionic spinal neuron-CG/SMG-gastrointestinal outflow (Figure 4*G*). Mice receiving AAV8-hSyn-DIO-mCherry served as controls. This system enabled precise manipulation of sympathetic activity in the gut (for detail, see[32]). Following CNO treatment, eosinophil count significantly increased in the colon of the hM3D group compared with that in the colons of control (mCherry) group mice (Figure 4*H*), indicating that activation of colon-innervating sympathetic nerves causes efficient recruitment of eosinophils to the colon.

NE Colorectal Infusion Induces Eosinophil Infiltration in the Colon of Both Mice and Rats

To further investigate the mechanisms involved in eosinophil recruitment by the SNS, we performed NE colorectal infusion and examined the distribution of immune cells in both mice and rats. Mice were employed in this study owing to the restricted accessibility of rat-specific antibodies required for flow cytometric analysis. Six hours after NE colorectal infusion, lamina propria cells were collected from the colon of mice for flow cytometry analysis. We observed specific increase in the percentage and the absolute cell number of eosinophils (relative to CD45^{+} cells) in the colonic tissue after NE infusion (Figure 5*A*–*C*). Notably, no significant increases were observed in other types of immune cells.

Consistently, immunohistochemistry (IHC) staining of rats revealed a significant increase in the number of MBP-ir cells after NE treatment, whereas neutrophils, macrophages, and mast cells showed no changes in the colon (Figure 6*A*, *B*). To investigate the role of adrenergic receptors in eosinophil infiltration, we treated rats with 2 pan-adrenergic receptor antagonists (phentolamine and propranolol) for 1

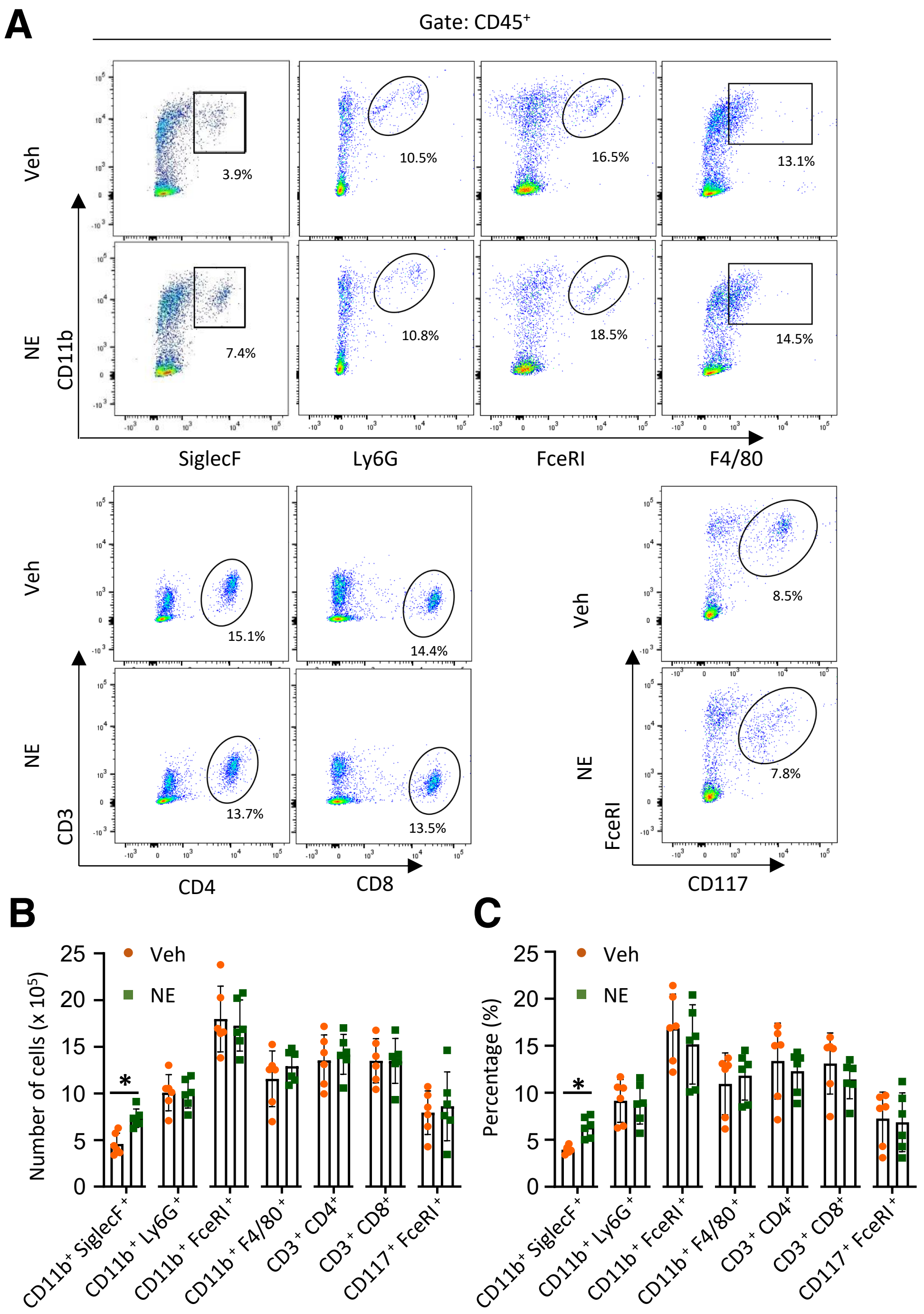

Figure 5. NE-colorectal infusion induces eosinophil infiltration in the colon of mice. (*A*) Flow cytometry analysis of immune cells gated in both Veh and NE-treated mice. (*B*, *C*) Bar graphs show the absolute cell number (*B*) and percentage (*C*) of immune cell alterations in the Veh- and NE-treated mice. (n = 6 in each group, percentage in CD45+ cells). Data are represented as mean ± SD. Statistical analysis was performed using the nonparametric Mann-Whitney *U* test. $^{*}P < .05$.

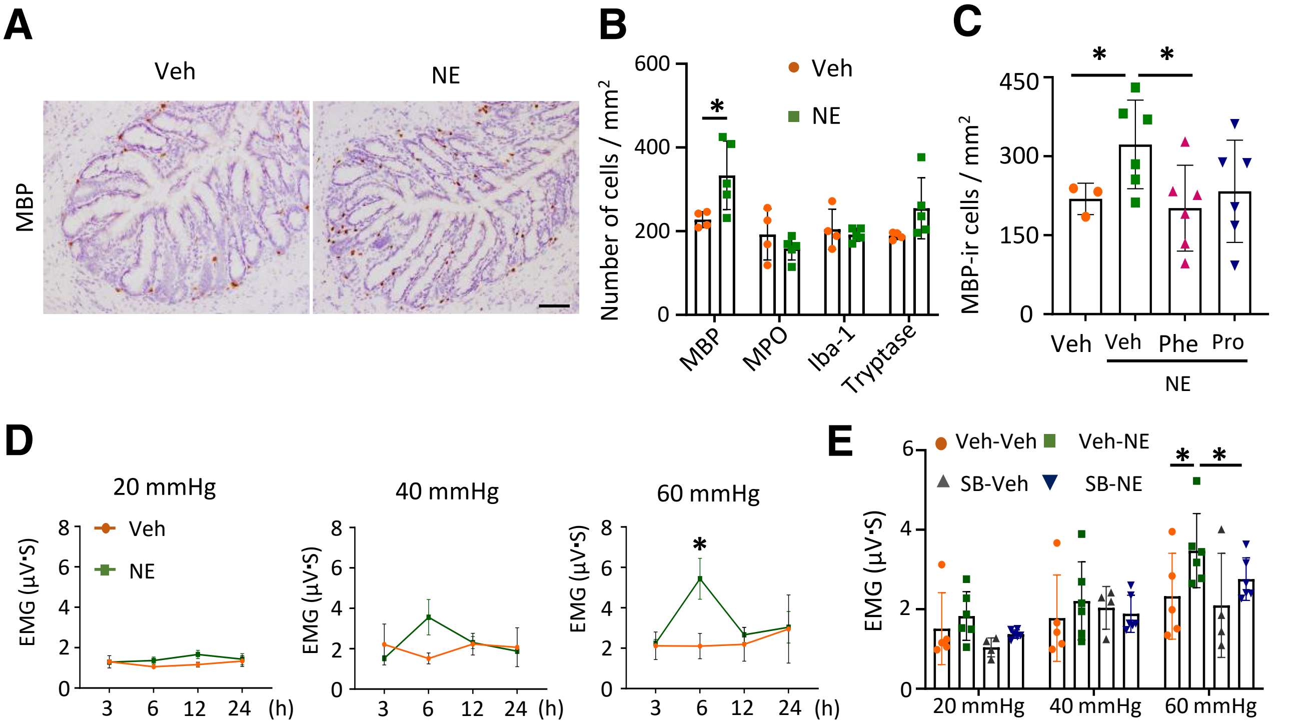

Figure 6. NE-colorectal infusion induces eosinophil infiltration and visceral pain of rats. (*A*) Representative images of IHC staining of MBP cells in colorectal tissue of Veh- and NE-treated rats. (*B*) Bar graphs show the numbers of MBP^+, MPO^+, Iba-1^+, and $tryptase^+$ cells in the colon of Veh- and NE-treated rats (n = 4–5 in each group). (*C*) The bar graph shows the number of MBP^+ cells with Phe and Pro administration in Veh- (n = 3) and NE-treated rats (n = 6 rats in each group). (*D*) Line graphs show EMG responses to CRD at different pressures (20, 40, and 60 mmHg) at 3, 6, 12, and 24 hours in Veh- and NE-treated rats. (*E*) The bar graph shows the EMG response to CRD at different pressures (20, 40, and 60 mmHg) in Veh/SB328437 treated rats with or without NE stimulation. (n = 4–6 in each group). Data are presented as mean ± SD. Nonparametric Mann-Whitney test was used for (*B*). One-way ANOVA was used in (*C*). Unpaired *t*-test was used for (*D*), and two-way ANOVA with Bonferroni post hoc test was used for (*E*). $^*P < .05$.

hour before NE infusion. Consistent with the findings observed in MS rats (Figure 1*K*), treatment with phentolamine significantly reduced eosinophil infiltration, whereas propranolol induced a trend towards a significant decrease in the number of eosinophils ($P = .12$). Meanwhile, there was no significant difference in MBP-ir cell count between the vehicle-treated and NE + propranolol-treated groups (Figure 6*C*). These results, along with the data from MS rats (Figure 1*K*), suggest that both alpha-AR and beta-AR play a role in regulating eosinophil migration in the colonic mucosa. To assess whether colonic adrenergic signaling induces visceral hypersensitivity and to determine its temporal dynamics, we measured the VMR to CRD at 3, 6, 12, and 24 hours following colonic NE infusion. A significant increase in VMR at 60 mmHg was observed at 6 hours postinfusion, indicating the onset of NE-induced colorectal hypersensitivity (Figure 6*D*).

Eotaxin-1 Mediates NE-induced Eosinophil Infiltration in Rats

As chemoattractants and cytokines play crucial roles in eosinophil recruitment to tissues,[33] we identified the specific cytokines and chemokines involved in NE-induced eosinophil infiltration in rats. We examined the expression of various genes (*Ccl11, Ccl24, Ccl26, Il33,* and *Il5*) associated with eosinophil infiltration after NE infusion into the colon of rats. Eotaxin-1 (*Ccl11*) was significantly upregulated in the colon 6 hours following NE infusion, whereas *Ccl24* and *Il33* exhibited no expression changes, and the expression of *Ccl26* and *Il5* was undetectable in the colonic mucosa (Figure 7*A, B*). Correspondingly, the expression of eotaxin-1-ir cells significantly increased from 3 hours after NE colorectal infusion (Figure 7*C, D*).

Eotaxin-1 can be released by epithelial cells, endothelial cells, macrophages, and mesenchymal cells.[34,35] To identify eotaxin-1 releasing cells involved after NE infusion, we performed double immunostaining of eotaxin-1 with CD68, vimentin, and CD31, representing markers of macrophages, mesenchymal cells, and endothelial cells, respectively (Figure 7*E*). Eotaxin-1 colocalized with vimentin and CD68 rather than CD31. Following NE treatment, the number of $vimentin^+eotaxin\text{-}1^+$ cells significantly increased compared with the Veh-treated group (Figure 7*F*). In contrast, the number of $CD68^+eotaxin\text{-}1^+$ cells did not differ between groups (Figure 7*F*). Colonic NE infusion did not alter the total number of $CD68^+$ cells or $vimentin^+$ cells (Figure 7*G*). These data suggest that NE colonic infusion upregulates eotaxin-1 predominantly in mesenchymal cells rather than other cell types.

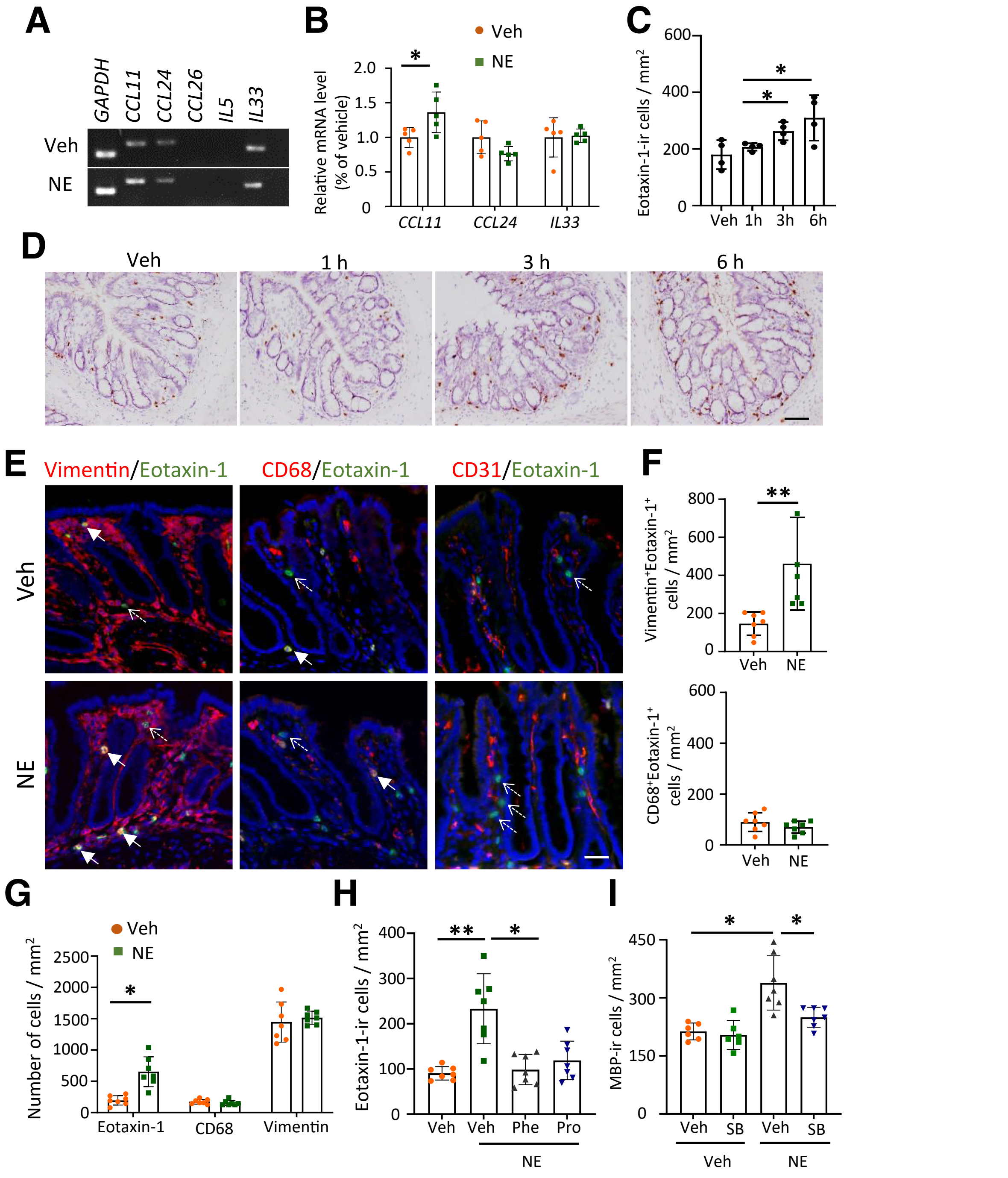

Figure 7. Eotaxin-1 contributes to the NE-induced eosinophil infiltration in the colon of rats. (*A*) PCR analysis of the expression of eosinophil recruitment genes (*Ccl11, Cccl24, Ccl26, Il5,* and *Il33*) in the colons of Veh and NE-treated rats 6 hours after NE treatment. (*B*) Bar graphs showing relative changes in gene expression between the colons of Veh- and NE-treated rats (n = 5 in each group). (*C*) Summary data of the number of eotaxin-1-ir cells at different time points after NE treatment. *$P < .05$, vs Veh (*D*). IHC images showing eotaxin-1 expression in the colon at 1, 3, and 6 hours following NE treatment. Scale bar: 50 μm. (*E*) Immunofluorescence images showing co-localization of eotaxin-1 with vimentin, CD68, and CD31 in the colons of Veh- and NE-treated rats, respectively. *Solid arrows* indicate double-labeled cells, and *dashed arrows* indicate single-labeled cells. Scale bar: 20 μm. (*F*) Bar graphs showing the number of vimentin^{+}eotaxin-1^{+} cells (*top*) or the CD68^{+}eotaxin-1^{+} cells (*bottom*) in the Veh- and NE-stimulated groups, respectively (n = 7 in each group). (*G*) Bar graphs showing data of eotaxin-1 cells, CD68 cells, and vimentin cells in the Veh- and NE-treated rats (n = 7 in each group). (*H*) Bar graphs showing the number of eotaxin-1-ir cells with or without Phe and Pro administration in Veh- and NE-treated rats (n = 7 in each group). (*I*) Bar graphs showing the number of MBP-ir cells with or without SB328437 administration in Veh- and NE-treated rats (n = 6 rats in each group). Data are presented as mean ± SD. A one-way ANOVA was used for the analysis shown in panel (*H*), a two-way ANOVA with Bonferroni post hoc test was used for the analysis shown in panel (*I*), unpaired *t*-tests were used for panels (*B*), (*F*), and (*G*), and the nonparametric Kruskal-Wallis test was used for panel (*C*) *$P < .05$, **$P < .01$. (Phe, phentolamine. Pro, propranolol).

To identify the adrenergic receptors involved in this regulation, we pretreated rats with antagonists of alpha-AR or beta-AR 1 hour before NE stimulation. Treatment with either the alpha-AR antagonist, phentolamine, or beta-AR antagonist, propranolol, inhibited the upregulation of eotaxin-1 (Figure 7*H*). Additionally, administration of the eotaxin-1 receptor inhibitor SB328437 significantly inhibited NE-induced eosinophil infiltration in the colonic mucosa (Figure 7*I*). These findings suggest that NE induces eosinophilic infiltration in the colon by upregulating eotaxin-1 in mesenchymal cells.

Furthermore, we ascertained whether chemogenetic activation of the SNS results in increased eotaxin-1 expression. The data from the selective activation of the SNS-gastrointestinal pathway in Cartpt-Cre mice infected with AAV8-hSyn-DIO-hM3D-mcherry demonstrated a significant increase in eotaxin-1 in the colon (Figure 4*I, J*). These findings suggest that SNS activity contributes to the increased eotaxin-1 expression.

NE Stimulates CCD-18co Cells to Release Eotaxin-1

Intestinal fibroblasts, the main components of prototypical mesenchymal cells, share the same immunophenotypic properties.[36] To confirm eotaxin-1 release from mesenchymal cells, we cultured CCD-18co cells, a non-malignant human fibroblast cell line that originates from normal colon tissue (Figure 8*A*). Stimulation with 10 mM NE induced significant upregulation of *hCCL11* from 3 hours onwards, whereas low concentrations and short treatment times did not affect the expression (Figure 8*B, C*). After NE stimulation, we detected a 13.8-fold increase in the protein level of eotaxin-1 in the culture supernatants at 3 hours via enzyme-linked immunosorbent assay (ELISA), which further increased to 35.7 times 6 hours after NE stimulation (Figure 8*D*). These data demonstrate that human intestinal fibroblasts can release eotaxin-1 after NE stimulation.

To examine whether colonic fibroblasts express ARs, we sorted mice colonic cells using flow cytometry. After excluding $CD45^+$ and $CD31^+$ cells, we identified $CD34^+$ $PDGFR\alpha^+$ cells as fibroblasts, based on previous research[37] (Figure 8*E*). Polymerase chain reaction (PCR) analysis of the sorted fibroblasts revealed the expression of α1B and β2 adrenergic receptors, consistent with the expression pattern observed in human CCD-18co cells (Figure 8*F*). These findings suggest that intestinal fibroblasts respond to NE stimulation by releasing eotaxin-1, thereby inducing eosinophilic infiltration into the colon.

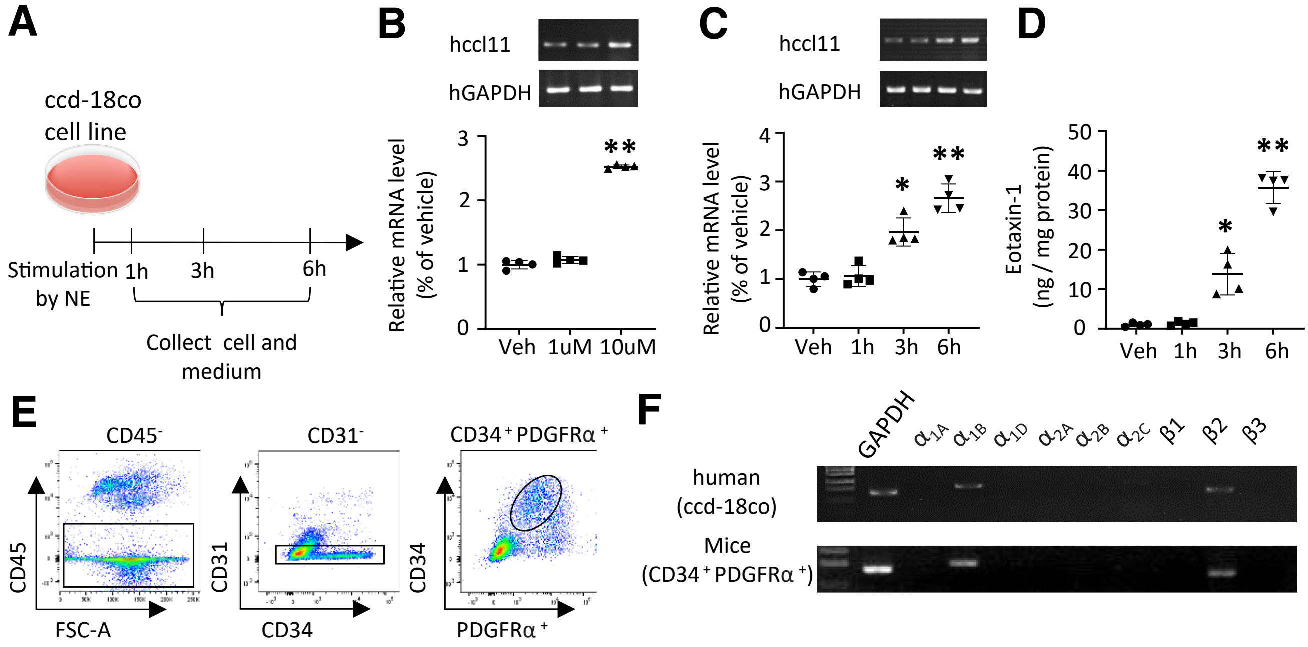

Figure 8. Eotaxin-1 is released from CCD-18co cells in response to NE stimulation. (*A*) The schematic diagram represents the protocols of NE treatment and sample collection. (*B*) Images and graphs show PCR bands and relative mRNA levels of *Hccl11* at different NE concentrations (Veh, 1 μM and 10 μM). (*C*) Images and graphs show PCR bands and relative mRNA levels of *Hccll11* at different time points (Veh, 1 hour, 3 hours, and 6 hours) under 10 μM NE stimuli. (*D*) The graph shows ELISA analysis of eotaxin-1 protein level in the CCD-18co cell culture serum at different time points (Veh, 1 hour, 3 hours, and 6 hours) under 10 μM NE stimuli. Data were collected from 4 independent experiments in (*B*), (*C*), and (*D*), respectively. (*E*) Graphs show the gating strategy of colonic fibroblasts in FACS. (*F*) Images show PCR bands of adrenoceptors expressing in sorted fibroblasts from the colon of human CCD-18co cell line (*top*) and the mice (*bottom*). Data are represented as mean $\pm$ SD. Statistical analysis was performed using the nonparametric Kruskal-Wallis test in panels (*B*), (*C*), and (*D*), with comparisons made against the respective Veh groups. *$P < .05$, **$P < .01$.

Eotaxin-1 Is Essential for Sympathetic Activity-induced Eosinophil Infiltration and Colorectal Hypersensitivity in the MS Model Rats

Having demonstrated that NE signaling modulates eosinophil infiltration via a mesenchymal cell-dependent mechanism in the colon, we subsequently examined whether the same underlying mechanism applies to the MS-induced IBS model rats. Eotaxin-1-ir cells were significantly increased in the colonic mucosa of MS rats but were reduced after 6-OHDA treatment (Figure 9*A*), supporting the role of sympathetic signaling. Additionally, 6-OHDA treatment suppressed visceral hypersensitivity in MS rats, as evidenced by a reduced VMR at 60 mmHg to CRD (Figure 9*B*).

To further investigate whether inhibition of eosinophil infiltration could attenuate visceral sensitivity in MS rats, dexamethasone (DEX) was administered for 3 consecutive days. This treatment significantly inhibited the eosinophil activation, as well as the visceral pain (Figure 9*C, D*).

As eotaxin-1 is the main chemoattractant causing eosinophil infiltration, we administered the eotaxin-1 receptor CCR3 antagonist SB328437 for 7 continuous days to both control and the MS rats and measured eosinophil infiltration and visceral response to CRD. Both visceral hypersensitivity and eosinophil infiltration were significantly suppressed in MS rats following SB328437 treatment (Figure 9*E, F*). In addition, visceral hypersensitivity induced by NE colorectal infusion was also significantly suppressed by SB328437 treatment (Figure 6*E*).

We also administered an eotaxin-1 colorectal infusion to directly evaluate its effect on eosinophil infiltration and visceral hypersensitivity. Eotaxin-1 infusion induced significant eosinophil infiltration into the colorectal mucosa (Figure 9*G*). Rats that received eotaxin-1 colorectal infusion exhibited hypersensitivity to CRD (Figure 9*H*).

These data collectively demonstrate that overactive sympathetic signaling results in eotaxin-1 release, ultimately inducing eosinophil infiltration and visceral hypersensitivity.

Upregulation of MBP- and Eotaxin-1-ir Cells in the Colorectal Tissue of Patients With IBS

Previous clinical studies suggest that a subset of patient with IBS show eosinophil infiltration in their GI tract.[6,38] To ascertain whether the presence of eotaxin-1-expressing cells, as observed in animal models, could also be identified in humans with IBS, biopsy specimens of colorectal tissue were obtained from both healthy controls and patients with IBS. We observed a pronounced elevation in the number of MBP-ir cells in the cecum or colorectal tissues of patients with IBS (Figure 10*A–D*). Although there was a trend toward an increased presence of tryptase-ir cells, no significant difference was observed between the groups (Figure 10*A–D*). Given the pivotal role of eotaxin-1 released from mesenchymal cells in eosinophil infiltration, we conducted double staining for eotaxin-1 and vimentin. Increased eotaxin-1-ir cells were observed in the colorectal tissues of patients with IBS, accompanied by an augmentation in the number of cells coexpressing vimentin, compared with those in the healthy controls (Figure 10*E, F*). Consequently, eotaxin-1 in vimentin-positive cells (which are likely to be fibroblasts) plays a pivotal role in the onset of eosinophil infiltration, which is a key factor in the pathogenesis of IBS.

Discussion

In the present study, using a rat model of MS stress, we successfully reproduced the main IBS characteristics, including immune alteration and visceral hypersensitivity, and confirmed that MS rats displayed sympathetic overactivity. Degeneration of sympathetic nerves by 6-OHDA reduced eosinophil infiltration in MS rats. Through chemogenetic approaches, we demonstrated that sympathetic activation induces eosinophil infiltration and visceral hypersensitivity in a mesenchymal cell-dependent manner in the colons of mice. Furthermore, patients with IBS showed upregulation of mesenchymal cells-derived eotaxin-1, associated with eosinophil infiltration in their colorectal tissue. These findings reveal that the SNS overactivation-induced release of mesenchymal cell-derived eotaxin-1 could be a potential inducer of eosinophil-associated mucosal immune alteration in patients with IBS.

Patients with IBS exhibit immune alterations in the GI.[5] Mast cells have been well-studied in both clinical and experimental animal research and implicated as important contributors to IBS-associated microinflammation. However, reports on mucosal mast cell counts are controversial, with some studies reporting upregulated, unchanged, or downregulated counts in patients with IBS.[8] Notably, a significant subset of patients with IBS does not exhibit mast cell alteration in the GI mucosa,[9,10,39–41] suggesting the involvement of additional mechanisms underlying microinflammation in IBS. Herein, no significant alterations were observed in the number of mast cells in the mucosal tissue in both the MS model and the sympathetic activation condition. Intestinal mast cells respond to various allergens, pathogens, and other agents that can be ingested, inhaled, or encountered after intestinal lumen epithelial barrier disruption.[42] The subtle alterations in mast cells might be attributed to slight changes in the luminal environment in MS rats.

Eosinophils are innate immune cells that play a significant role in the GI and maintain intestinal homeostasis.[33] Clinical studies have reported that a subpopulation of patients with IBS exhibits colonic eosinophil infiltration and activation.[4–6,11,12,38] Our observation of colonic eosinophil infiltration may therefore be relevant to these clinical findings, although further investigations are required to establish a direct relationship.

Eosinophil proliferation, maturation in the bone marrow, and migration to the circulation are regulated by interleukin (IL)5 released from ILC2 cells.[34,43] The recruitment of eosinophils to the GI is regulated primarily by eotaxin-1. We observed that IL5 expression did not change in mucosa, whereas expression of the eotaxin-1

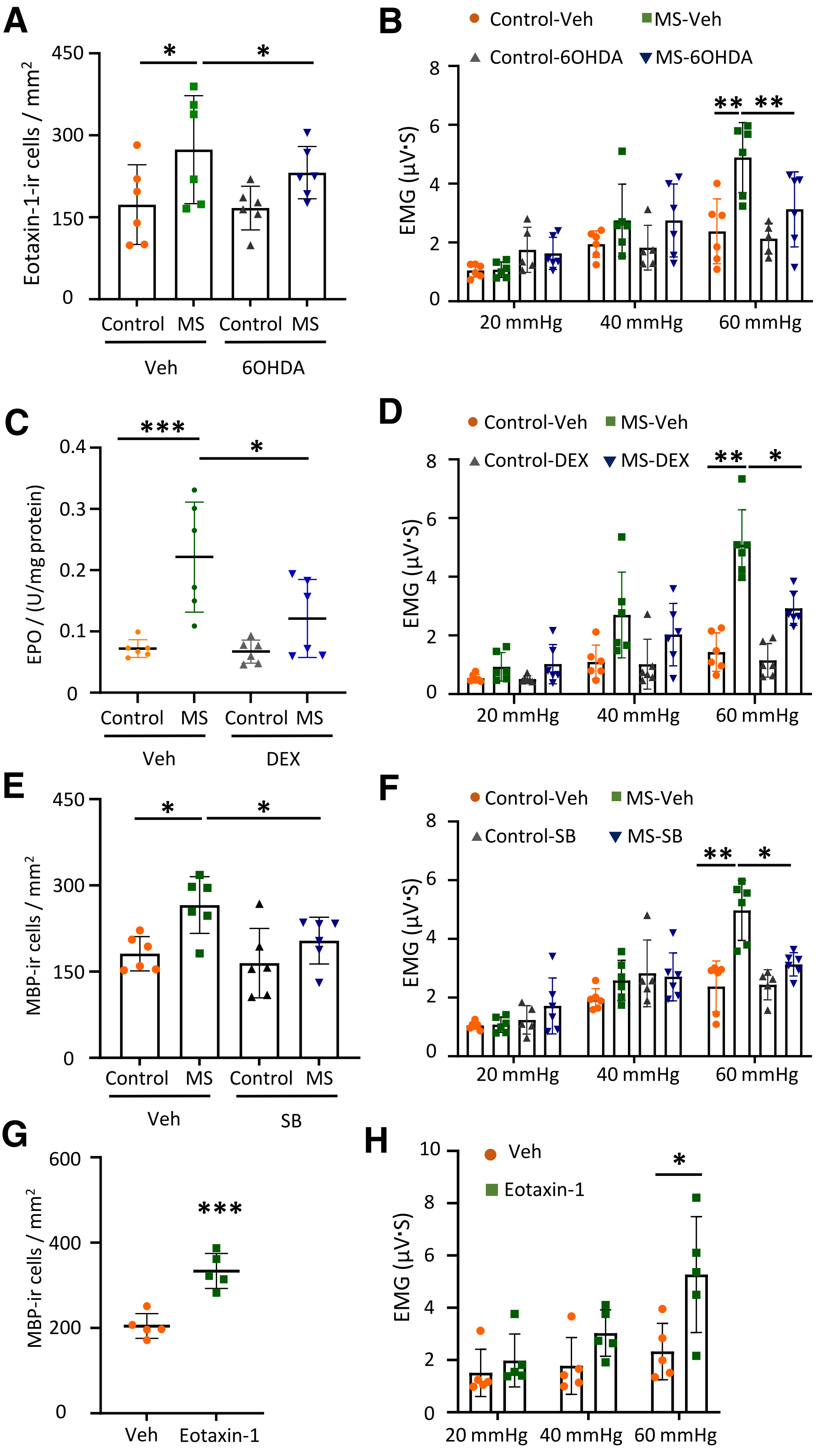

Figure 9. Eotaxin-1 is essential for eosinophil infiltration and colorectal hypersensitivity in the MS model rats. (*A*) Bar graphs show numbers of eotaxin-1-ir cells in control and MS rats with/without 6-OHDA treatment (n = 6 in each group). (*B*) Bar graphs represent the data of VMR response to CRD in control and MS rats with/without 6-OHDA treatment (n = 6 in each group). (*C*) Bar graph showing EPO activity in control and MS rats treated with Veh or DEX (n = 6 in each group). (*D*) Bar graph showing the VMR to CRD in control and MS rats treated with Veh or DEX (n = 6 in each group). (*E*) Bar graph shows the numbers of MBP-ir cells in the control and MS groups with/without SB328437 treatment (n = 6 in each group). (*F*) Bar graphs represent data of EMG response to CRD in control and MS rats with Veh/SB328437 treatment (n = 6 in each group). (*G*) The graph shows the number of MBP-ir cells following eotaxin-1 treatment (n = 5 in each group). (*H*) Bar graphs represent EMG response to CRD following Veh/eotaxin-1 (n = 5 in each group). Data are represented as mean ± SD. Statistical analysis was performed using two-way ANOVA with Bonferroni post hoc test for panels (*A–F and H*), and the nonparametric Mann-Whitney test for panel (*G*). *$P < .05$, **$P < .01$, ***$P < .001$.

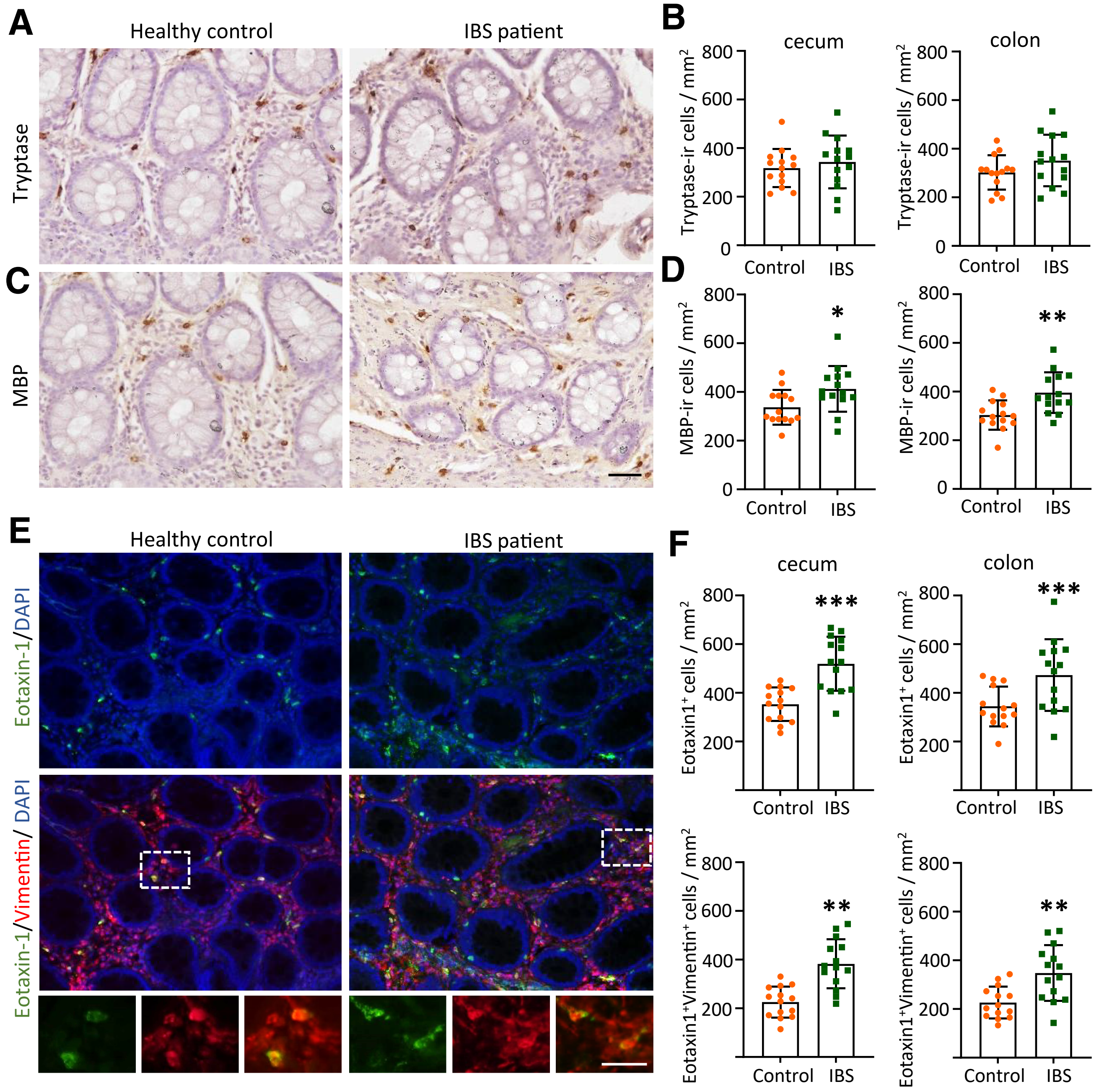

Figure 10. Upregulation of MBP- and eotaxin-1-ir cells in colorectal tissues of patients with IBS. (*A–B*) Representative images of tryptase immunoreactivity (*A*) and the number of tryptase-ir cells (*B*) in the cecum and colon of healthy controls and patients with IBS. (*C–D*) Representative images of MBP immunoreactivity (*C*) and the number of MBP-ir cells (*D*) in the cecum and colon of healthy controls and patients with IBS. Scale bar: 20 μm. (*E*) Representative images of eotaxin-1 and vimentin in healthy controls and patients with IBS. (*F*) Number of eotaxin-1-ir cells and eotaxin-1^{+}vimentin^{+} cells in the cecum and colon of controls and patients with IBS (n = 14 in each group). Data are presented as mean ± SD. Unpaired *t*-test was used. $^{*}P < .05$.

gene increased in the intestinal mucosa after NE stimulation or in MS rats. This result suggests that sympathetic activation induces eosinophil infiltration via the eotaxin-1 pathway rather than the ILC2 pathway.

In healthy individuals, eotaxin-1 is responsible for the normal recruitment of eosinophils to the GI and is continuously expressed in the intestinal lamina propria.[35] Eotaxin-1 deficient mice exhibit a large, selective reduction in eosinophils residing in the intestinal tract.[35] Eotaxin-1 is produced by various cell types, including macrophages, fibroblasts, and epithelial cells.[44] We found that in the intestinal mucosa, eotaxin-1 was mainly expressed by macrophages and vimentin-positive mesenchymal cells but not by endothelial cells. After NE stimulation, the number

of mucosal eotaxin-1-expressing mesenchymal cells (but not macrophages) increased, indicating that mesenchymal cells are the main source of eotaxin-1 in response to sympathetic signalling. These vimentin-positive mesenchymal cells in the intestinal mucosa were defined as lamina propria fibroblasts or myofibroblasts. Moreover, both the human fibroblast cell line and mice colonic fibroblasts expressed α1B and β2 adrenergic receptors (Figure 8*F*). These data suggest that intestinal fibroblasts are regulated by sympathetic signalling and play a crucial role in eosinophil migration and infiltration through the release of eotaxin-1. In our study, both the findings from animal models and human samples suggest that fibroblasts releasing eotaxin-1 played a role in the eosinophil infiltration through sympathetic signaling rather than antigen triggers. Our findings highlight a novel mechanism by which fibroblasts participate in IBS pathogenesis and suggest potential therapeutic targets for this complex disorder.

Fibroblasts constitute a crucial component of the GI stroma and significantly contribute to wound healing, extracellular matrix remodeling, and immune responses. In inflammatory conditions such as IBD, fibroblasts act as major sources of inflammatory cytokines and chemokines, perpetuating chronic tissue inflammation.[45,46] The current observations suggest that fibroblasts can be regulated by the adrenergic signalling, thereby offering a novel underlying mechanism and potential therapeutic approach for these diseases or conditions.

Visceral pain is a key pathological feature of IBS, which involves the central and peripheral nervous systems.[47,48] Herein, we demonstrated that eosinophil infiltration contributed to colorectal hypersensitivity in an MS stress-induced IBS model. Activation of the SNS by microinjecting AAV2-hSyn-DIO-hM3D-mCherry into the CG-SMG, followed by CNO stimulation, induced visceral pain in response to colorectal distension. This finding suggests that continuous CNO stimulation may promote broader eosinophil infiltration and activation, thereby enhancing the sensitivity of the entire colorectum to distension.

In humans, the colorectum is known to receive sparse innervation from the CG-SMG. However, as a limitation of our study, we were unable to directly assess the response of the proximal colon to distension, to avoid injuring the animals by inserting the balloon too deeply into the colon. To verify that AAV-infected fibers potentially innervate the distal colon in mice, we examined the presence of mCherry-immunoreactive fibers in the distal colon, 1 to 3 cm from the anus, and identified a subset of such fibers in this region (Figure 4*B*).

In the current study, we did not focus on the mechanisms of which eosinophil infiltration leads to visceral hypersensitivity. Eosinophils can release various bioactive mediators, such as MBP, EPO, cationic proteins, neuropeptides, and reactive oxygen species. These mediators may interact with receptors in the peripheral afferent, resulting in diverse neuronal effects such as neural plasticity, nerve damage, and hypersensitivity conditions.[29,49] The molecular and cellular mechanisms underlying eosinophil-induced colonic sensory hypersensitivity remain to be fully elucidated. Additionally, it is important to recognize that visceral sensory signals are also processed within the enteric nervous system (ENS), particularly in the myenteric and submucosal plexuses, which have been implicated in the pathophysiology of IBS.[50] Overactivation of the SNS may modulate the sensitivity of enteric neuron or glia, thereby contributing to the immune alterations associated with visceral pain.[51,52] Therefore, although our findings provide insights into mucosal immune responses, they should be interpreted within the broader context of ENS involvement in gastrointestinal sensory processing and the potential impact of SNS modulation on these pathways.

Our study focused on investigating the role of overactive peripheral SNS on immune alteration using an IBS animal model. The results suggest a potential mechanism underlying eosinophil infiltration and visceral hypersensitivity. Nevertheless, we acknowledge the contribution of HPA axis dysregulation in the IBS pathogenesis and the contribution of mast cells to the immune dysregulation of IBS. We believe both mast cells and eosinophils contribute to intestinal microinflammation in the complex pathogenesis of IBS. As only male animals were used in the present study, future investigations including both sexes will be necessary to determine whether these findings are generalizable across sexes.

Conclusion

The present study revealed that SNS overactivation led to eosinophil infiltration in the colon of the MS stress-induced IBS model. This infiltration is due to the release of eotaxin-1 from the mesenchymal cells, contributing to the development of visceral hypersensitivity. Our findings not only suggest that fibroblast-derived eotaxin-1 and eosinophil could be potential targets for therapeutic interventions in the treatment of IBS but also provide evidence for understanding the effectiveness of psychotherapy, which can relieve sympathetic activity in IBS management.

Materials and Methods

Animals and Study Approval

B6.Cg-Tg (DBH-Cre) 9-9Koba/KobaRbrc (RBRC01492) mice were acquired from Professor Kobayashi[53] through the RIKEN BRC of the National BioResource Project of MEXT/AMED, Japan. Cartpt-Cre mice (accession No. CDB0267E) lines (listed in https://large.riken.jp/distribution/mutant-list.html) were generated via CRISPR/Cas9-mediated knock in zygotes as reported previously.[32] Sprague-Dawley rats that were 14 days pregnant and adult (7 weeks old) were purchased from Japan SLC Inc and housed in a standard laboratory with free access to water and food. Male litters were used in the study to avoid the potential confounding effects of estrous cycle-related hormonal fluctuations. Different animal species were used for specific experiments to take advantage of species-specific techniques. DREADD-based neuronal manipulation was performed in DBH-cre mice and Cartpt-cre mice,

whereas other physiological or histological analyses were conducted in rats due to established model availability. The species used for each experiment are explicitly indicated throughout the results and figure legends.

The animals in the study were housed and fed under the exact same conditions. Following the 3R principles, the experimental protocols were designed to minimize the number of animals used. The number of animals used in each group is mentioned separately in the figure legends. The experimental protocols were designed to minimize the number of animals used and were approved by the Committee on Animal Research at Hyogo Medical University (#2021-22, 2021-18, and 2021-04).

Human Endoscopic Biopsy Specimens

Patients diagnosed with IBS were identified based on the Roma IV criteria.[1] None of the participants had taken any anti-inflammatory medications. Cecum and colon biopsy specimens were obtained from 14 healthy controls (n = 7 for female, n = 7 for male) and 14 patients (n = 4 for female, n = 10 for male, IBS-C n = 1, IBS-D n = 12, IBS-M n = 1) during endoscopy. These biopsy samples were used for IHC staining. All patients with IBS and the controls were recruited from Hyogo Medical University Hospital. This study was performed in accordance with the Declaration of Helsinki and was approved by the Ethics Committee/Institutional Review Board of Hyogo Medical University (No. 4706). All participants provided written informed consent. Patients were not involved in the design, or conduct, or reporting, or dissemination plans of our research.

MS Model

The MS rat model was established as described previously.[28] Briefly, newborn pups were randomly divided into the control and MS groups. MS pups were individually placed in disposable paper cups (5 cm in diameter) for 3 hours per day for 14 days. After separation, the pups were returned to their mother's cage, whereas the control pups remained with their mothers continuously. The rats were reared for up to 7 weeks for subsequent experiments.

Electrode Implantation

Electrodes were implanted into the external oblique muscles of the rats or mice to record muscle contraction, as reported previously.[28] Surgery was performed under 2% isoflurane-induced general anaesthesia. Two wires (Cooner Wire) were implanted into the left external oblique muscle, and one wire was implanted on the other side. Wires were externalised at the backs of the animals, and the skin was closed with a suture. The animals were allowed 5 days to recover before the next procedure.

Colorectal Distention and Electromyography Recording

To allow for calm CRD in the awake state, animals were trained to acclimate to a cylindrical box (rat: 15 cm length $\times$ 5 cm diameter; mouse: 7 cm length $\times$ 3 cm diameter). CRD was performed using a slack latex balloon (Okamoto), inserted 4 cm from the anus in rats and 1 cm in mice. The ballon was attached to a gavage needle and inserted into the colon through the anus. Varying pressure (20, 40, and 60 mmHg) was applied to the colon by inflating the balloon with 37°C water. Each pressure was tested for 20 seconds at a 5-minute interval time. The VMR to CRD was recorded using electromyography (EMG) with wires implanted in the muscle. The EMG signals were filtered at 1000 Hz and recorded using Power Lab (AD Instruments). The area under the curve (AUC) was calculated for 10 seconds at baseline and during CRD to minimize potential variability occurring at the end of the stimulation period. The VMR at each CRD pressure was normalized to the baseline. The threshold was calculated from a ramp distention experiment independently, and the pressure of the CRD at which the VMR increased to 5 times the baseline value was characterized as the threshold.

AAV

The plasmid of capsid AAV PHP.S, pUCmini-iCAP-PHP.S (Addgene, Plasmid No. 103006), AAV Helper plasmid (Takara), and shuttle plasmid, and AAV-hSyn-DIO-hM3D-mCherry (Addgene, Plasmid No. 44361) were transfected in HEK293T cells (RIKEN BRC). Virus particles were purified via OptiPrep ultracentrifugation, concentrated using Amicon Ultra-15 centrifugal filter unit (Millipore), and kept in 1 $\times$ phosphate buffered saline (PBS). Titre was measured via quantitative PCR with SYBR Green (Takara).

AAV2 hSyn-DIO-hM3D-mcherry (Addgene #44361), AAV8 hSyn-DIO-hM3D-mCherry (Addgene #44361) and AAV8 hSyn-DIO-mCherry (Addgene #50459) vectors were obtained from Addgene.

AAV Infection

Retro-orbital injection. Four-week-old DBH-Cre mice were anesthetised with 2% isoflurane (Wako Pure Chemical Corporation). The mouse was positioned on its side, and the loose skin around the eyes was pulled back using the fingers. The eye protruded slightly, and a 30-G needle of an insulin syringe (BD) was inserted at an angle of approximately 45°. The needle was passed through the inferior conjunctiva into the retroorbital sinus.[54] A 150 μL dose of AAV PHP.S-hSyn-DIO-hM3D-mCherry (1×10^{13} viral genomes/mL) was injected into the sinus, and the needle was gently removed to prevent eye damage. The next procedure was performed 4 weeks after AAV injection.

CG-SMG injection. Four-week-old DBH-Cre mice were anesthetized with 2% isoflurane. Abdominal incision was made, and the intestines were gently exteriorized to expose the CG-SMG. A glass micropipette containing 2 μL of AAV2 hSyn-DIO-hM3D-mcherry vector mixed with 0.1% Fast Green (#F7252, Sigma-Aldrich) was inserted into the CG-SMG, and the vector was microinjected.

Spinal cord injection. An incision was made around the thoracic region of the spinal cord in Cartpt-Cre mice. To expose the spinal cord, the paraspinal muscles and dorsal vertebral elements were carefully excised using fine forceps. A glass pipette was utilized to administer 100 nL of the AAV8 hSyn-DIO-hM3D-mCherry vector with 0.1% Fast Green at 10 distinct sites (infusion rate, 60–70 nL/min) bilaterally.

Colon Lamina Propria Cell Harvest and Flow Cytometry

Colon tissues of wild-type mice 6 hours after NE colorectal infusion and those of DBH-Cre mice after 5 days of continuous CNO/saline stimulation were collected under 2% isoflurane anaesthesia. Samples were placed in cold Hank's balanced salt solution (HBSS), and fecal pellets, blood vessels, and mesenteric tissue were carefully removed. The samples were cut along the longitudinal axis into 1-cm pieces and placed in HBSS containing 1 mM dithiothreitol-ethylenediaminetetraacetic acid and 10% fetal bovine serum (FBS). The samples were shaken at 37°C and 150 rpm for 20 minutes to remove the epithelial cells. The samples were then placed in Roswell Park Memorial Institute 1640 medium (Nacalai), containing 10% FBS, 1 mg/mL Collagenase IV (Sigma-Aldrich), and 1 mg/mL Dispase II, and shaken for 60 minutes to obtain a single-cell solution. The single-cell solution was centrifuged through discontinuous Percoll (Cytiva) gradients (40%: 80%) to collect cells from the lamina propria. The collected cells were washed with HBSS, and the cell density was adjusted to 1×10^6 cells/mL.

The cells were resuspended in HBSS containing 10% FBS and incubated with fluorescently conjugated antibodies for 30 minutes on ice. The following fluorescently conjugated antibodies were purchased from BioLegend and used: PE/Cy7 anti-mouse CD45 (30-F11, 1:500), FITC anti-mouse CD11b (M1/70, 1:200), PE anti-mouse Siglec-F (S17007L, 1:200), PerCP/Cy5.5 anti-mouse FceRIa (MAR-1, 1:200), APC anti-mouse Ly-6G (1A8, 1:200), BV711 anti-mouse F4/80 (BM8, 1:200), BV711 anti-mouse CD117 (2B8, 1:200), FITC anti-mouse CD3 (145-2C11, 1:200), PE anti-mouse CD4 (GK1.5, 1:200), and APC anti-mouse CD8 (53-6.7, 1:200); 4',6-diamidino-2-phenylindole (DAPI, 1:1000) was also purchased. After washing the cells with PBS containing 0.2% bovine serum albumin (BSA), the fluorescence intensity of individual cells was determined using a FACSAriaIIIu flow cytometer (BD Bioscience). Dead cells were stained with DAPI and excluded from the analysis. For analysis, the adhesion signals were excluded by FSC-A(area) and FSC-H (height) at first, and then approximately 50,000 events were gated to live cells using DAPI in each sample. Subsequently, the gates were set on anti-CD45 positive events, followed by immune cell gating separately. FlowJo software (BD Bioscienes) version 10.8.1 was used for data analysis.

Sorting of CD34+ PDGFRα+ Cells

A single-cell suspension from the colon lamina propria was prepared as described above. Cells were resuspended in HBSS containing 10% FBS and incubated with fluorescently conjugated antibodies for 30 minutes on ice. The following fluorescently conjugated antibodies were purchased from BioLegend and used: APC anti-mouse CD34 (MEC14.7, 1:200), PE anti-mouse PDGFRα (APA5, 1:200), FITC anti-mouse CD31 (390, 1:200), and PE/Cy7 anti-mouse CD45 (30-F11, 1:500). After washing and resuspending in HBSS containing 2% FBS, mouse fibroblasts (CD45- CD31- CD34+ PDGFRα +) were sorted via BD FACSAriaIIIu (BD Biosciences). Endothelial and immune cells were excluded as CD31- and CD45-positive cells, respectively.

IHC

IHC was performed as described previously.[28] Briefly, rat and mice were anesthetised with 2% isoflurane and transcardially perfused with 1% paraformaldehyde, followed by 4% paraformaldehyde. The brain, superior cervical ganglion, DRG, spinal cord, and GI tract (rat: 3–5 cm from anus; mouse: all colon tissue) tissues were dissected and post-fixed in 4% paraformaldehyde for 24 hours at 4°C. The tissues were then transferred to 30% sucrose for 2 days and finally embedded in Tissue-Tek O.C.T. (Sakura Finetek). In a parallel experiment, fresh tissues were dissected without paraformaldehyde perfusion and embedded in Tissue-Tek OCT. Frozen samples were sectioned at 10-μm thickness using a cryostat (Thermo Fisher Scientific) and prepared for IHC staining. Fresh sections were post-fixed using acetone. Human paraffin sections were first incubated with 0.4% pepsin for 20 minutes at 37°C and then with primary antibodies overnight at 4°C, followed by secondary antibody incubation for 1 hour at room temperature. The primary and secondary antibodies used in this study are listed in Supplementary Tables 1 and 2. As reported previously, signals were obtained via immunofluorescence or 3, 3′-diaminobenzidine (DAB) staining.[28] The validity and unspecific binding of antibodies were assessed by nonprimary controls. Sections were observed under a microscope (Nikon, Eclipse 80i, Nikon Instruments), and images were taken at 10× or 20× magnification using NIS-Elements D 3.2 software. Three tissue areas for analyses were randomly taken from one section. Three sections were included for each animal, and 4 to 6 animals were included in each group. Positive cells were visually count in a blinded manner (in chemo genetic and human sample analysis). Cells counts were analyzed using the ImageJ and Fiji software.

Hematoxylin and Eosin Staining

Ten-μm thick sections of colon tissue were prepared as described above. The sections were stained with Mayer's hematoxylin (Sigma) for 2 minutes followed by eosin (Sigma) for 15 seconds. Subsequently, the sections were dehydrated and sealed.

Double Staining

To exclude nonspecific immunofluorescence signals in the colonic samples, we performed double IHC of certain

antibodies with DAB combined with alkaline phosphatase (AP) staining. Incubation with primary antibodies was performed as described above. The sections were then incubated with horseradish peroxidase-conjugated or AP-conjugated secondary antibodies separately for 1 hour at room temperature. Horseradish peroxidase was detected using DAB staining as previously described.[28] AP was detected using the ImmPACT Vetor Red AP Substrate Kit (SK-5105; Vector) according to the manufacturer's instructions.

Mouse-on-mouse Staining

We used a mouse anti-cre antibody from Sigma-Aldrich to detect DBH-Cre, MBP, and eotaxin-1-positive cells. MOM immunodetection kit (BMK-2202; Vector) was used for staining according to the manufacturer's instructions.

ELISA

Eotaxin-1 ELISA. CCD-18Co cells were cultured in 6-well plates at a density of 5×10^4 /mL and incubated at 37°C under 5% CO_2 before NE stimulation. The medium was replaced with 0, 1, and 10 μM NE and incubated for 1, 3, or 6 hours. After incubation, the supernatants were harvested for eotaxin-1 ELISA assay using the Human CCL11/Eotaxin Kit (R&D Systems, Inc) according to the manufacturer's protocol.

NE ELISA. Blood samples were collected from the veins of adult MS rats or DBH-Cre mice after 5 days of continuous CNO/saline stimulation under 2% isoflurane anesthesia and kept for 6 hours at 4°C to clot. The clot was removed by centrifugation at 1500 g and 4°C for 10 minutes. The supernatants were subjected to NE ELISA (LifeSpan Biosciences, Inc) according to the manufacturer's instructions.

Corticosterone ELISA. Blood samples were collected from the veins of DBH-Cre mice infected with AAV2-hSyn-DIO-hM3D-mCherry after 5 continuous days of CNO or saline treatment. Serum was isolated 6 hours after clotting and subjected to corticosterone quantification using an ELISA kit (Yanaihara), according to the manufacturer's instructions.

RT-PCR

CCD-18Co cells. CCD-18Co cells (American Type Culture Collection) were cultured in 6-well plates as described above. The cells were stimulated with 0, 1, and 10 μM NE and incubated for 1, 3, or 6 hours. After incubation, the cells were collected for PCR analysis.

Sorted cells. $CD34^+$ $PDGFR\alpha^+$ sorted by FACS were collected in a tube; total RNA extraction and PCR protocols were described below.

Colonic tissue. Six hours after NE colorectal infusion, rats were anesthetised with 2% isoflurane. The colon tissue was dissected and rapidly frozen on dry ice. The colonic tissues were dissected from control and MS model rats at 7 to 8 weeks old and frozen on dry ice. Total RNA was extracted, and PCR was performed as below.

Bone marrow. Bone marrow was dissected from the hind limb long bones (femurs) from the control or MS rat after sacrifice. Total RNA was extracted, and PCR was performed as below.

Total RNA was extracted using TRIzol (Sigma-Aldrich) following the instruction. A total of 5 μg of RNA was combined with 25 μL of reverse transcription (RT) mixture, which included 200 U of M-MLV reverse transcriptase (Promega), 20 U of RNase inhibitor (Promega), 0.8 μM dNTP, 1 μg oligo-dT primer, and 1× reaction buffer (pH 7.5; Promega). The RT reaction was conducted at 37°C for 60 minutes, followed by enzyme inactivation at 65°C for 10 minutes. Subsequently, PCR was performed in a 20-μL reaction using the Taq PCR Master Mix Kit (Qiagen), following the manufacturer's protocol. PCR conditions were set for 30 to 32 cycles, each consisting of denaturation at 94°C for 15 seconds, annealing at 60°C for 15 seconds, and extension at 72°C for 45 seconds. The amplified products were separated by electrophoresis on a 1.5% agarose gel, then reaction with ethidium bromide for 30 minutes and visualized under UV light.

The primers used in the reverse transcription-PCR are listed in Supplementary Table 3.

Colorectal Infusion

The rats or mice used for colorectal infusion experiments were anesthetised with 2% isoflurane, and a plastic oral gavage needle was slowly inserted into the colon through the anus. The needle was inserted 2 cm from the anus, and NE or saline was injected slowly to avoid leakage. After the injection, the animals were allowed to keep their hips elevated for 10 min. Norepinephrine (Sigma-Aldrich) was injected at 0.5 mg/kg. The next steps were performed 6 hours after NE. Eotaxin-1 (PeproTech) was injected at 1 mg/kg for rats, and EMG was recorded 24 hours after the injection. DEX (1mg/kg in 0.3% DMSO) was infused to MS/control rats once daily for 3 consecutive days prior to experimentation.

EPO Assay

EPO activity was tested as our previous reports.[28] 0.5 cm-long colon tissues were taken from rats after sacrifice. Colon of DBH-Cre mice infected with AAV2-hSyn-DIO-hM3D-mCherry were collected after CNO stimulation. For the EPO assay, tissue samples were homogenized in 50 mM Tris·HCl (pH 8.0) with 0.1% Triton X-100 at a ratio of 1 mL per 50 to 100 mg of tissue. The homogenate was centrifuged at 1600 g for 5 minutes at 4°C. The resulting supernatants were used to measure EPO activity, utilizing the oxidation of o-phenylenediamine (OPD) in the presence of H_2O_2. For the assay, 20 μL of the supernatant was combined with 580 μL of 50 mM Tris·HCl buffer containing 10 mM OPD (Nacalai) and 4 mM H_2O_2. Enzyme activity was determined photometrically at 492 nm following a 5-minute incubation. The protein concentration was assessed by the Bio-rad protein assay dye reagent (Bio-rad) concentrate following the instruction of the products.

Fecal Pellets Analysis

After intraperitoneal injection of CNO or vehicle, DBH-Cre mice were placed in a clean cage for 2 hours for fecal pellet collection. The number of fecal pellets in the clean cage was counted, and the data were recorded once a day for 5 days. Data are presented as the number of pellets formed per hour.

6-OHDA Treatment

To degenerate SNS activity, 6-OHDA (Cayman Chemical) was dissolved in saline containing 1% ascorbic acid. Seven-week-old rats (MS and control) were intraperitoneally administered with 6-OHDA (150 mg/kg). The vehicle group was administered the same solution but without 6-OHDA. Subsequent experiments were performed 7 days after 6-OHDA treatment.

Pharmacologic Agents

CNO (Cayman Chemical) was dissolved in saline containing 0.5% dimethylsulphoxide (DMSO) and administered intraperitoneally (1 mg/kg) for 5 continuous days. The eotaxin-1 CCR3 receptor antagonist SB328437 (Tocris Bioscience) was intraperitoneally injected at a dose of 1 mg/kg with 10% DMSO and 0.1% Tween 20. The pan-alpha AR antagonist phentolamine (R&D Systems; 1 mg/kg in saline) and the pan-beta AR antagonist propranolol (pro) (R&D Systems; 10 mg/kg in saline) were administered intraperitoneally. Before experiments, the MS model was treated with SB328743, phentolamine, and propranolol for 7 continuous days and 1 hour before the NE colorectal infusion. The treatment of each group was finished within short time intervals to minimize potential confounders.

Data Analysis

Statistical analyses were performed using Microsoft Excel and GraphPad Prism 9. The statistical methods used in the study are indicated in the figure legends. Nonparametric tests (Mann-Whitney U test or Kruskal-Wallis test) were used to compare groups when data did not follow a normal distribution. All data are reported as mean ± standard error (SE). P-values $< .05$ were considered statistically significant ($^*P < .05$, $^{**}P < .01$, and $^{***}P < .001$).

Supplementary Material

NOTE: To access the supplementary material accompanying this article, visit the full text version at https://doi.org/10.1016/j.jcmgh.2025.101658.

References

1. Drossman DA, Hasler WL. Rome IV-functional GI disorders: disorders of gut-brain interaction. Gastroenterology 2016;150:1257–1261.
2. Fukudo S, Okumura T, Inamori M, et al. Evidence-based clinical practice guidelines for irritable bowel syndrome 2020. J Gastroenterol 2021;56:193–217.
3. Camilleri M. Peripheral mechanisms in irritable bowel syndrome. N Engl J Med 2012;367:1626–1635.
4. Lazaridis N, Germanidis G. Current insights into the innate immune system dysfunction in irritable bowel syndrome. Ann Gastroenterol 2018;31:171–187.
5. Burns G, Carroll G, Mathe A, et al. Evidence for local and systemic immune activation in functional dyspepsia and the irritable bowel syndrome: a systematic review. Am J Gastroenterol 2019;114:429–436.
6. Katinios G, Casado-Bedmar M, Walter SA, et al. Increased colonic epithelial permeability and mucosal eosinophilia in ulcerative colitis in remission compared with irritable bowel syndrome and Health. Inflamm Bowel Dis 2020;26:974–984.
7. Salvo-Romero E, Martinez C, Lobo B, et al. Overexpression of corticotropin-releasing factor in intestinal mucosal eosinophils is associated with clinical severity in diarrhea-predominant irritable bowel syndrome. Sci Rep 2020;10:20706.
8. Bashashati M, Moossavi S, Cremon C, et al. Colonic immune cells in irritable bowel syndrome: a systematic review and meta-analysis. Neurogastroenterol Motil 2018;30:e13192.
9. Chang L, Adeyemo M, Karagiannides I, et al. Serum and colonic mucosal immune markers in irritable bowel syndrome. Am J Gastroenterol 2012;107:262–272.
10. Braak B, Klooker TK, Wouters MM, et al. Mucosal immune cell numbers and visceral sensitivity in patients with irritable bowel syndrome: is there any relationship? Am J Gastroenterol 2012;107:715–726.
11. Park KS, Ahn SH, Hwang JS, et al. A survey about irritable bowel syndrome in South Korea: prevalence and observable organic abnormalities in IBS patients. Dig Dis Sci 2008;53:704–711.
12. De Silva AP, Nandasiri SD, Hewavisenthi J, et al. Subclinical mucosal inflammation in diarrhea-predominant irritable bowel syndrome (IBS) in a tropical setting. Scand J Gastroenterol 2012;47:619–624.
13. Aguilera-Lizarraga J, Hussein H, Boeckxstaens GE. Immune activation in irritable bowel syndrome: what is the evidence? Nat Rev Immunol 2022;22:674–686.
14. Posserud I, Agerforz P, Ekman R, et al. Altered visceral perceptual and neuroendocrine response in patients with irritable bowel syndrome during mental stress. Gut 2004;53:1102–1108.
15. Schaper SJ, Stengel A. Emotional stress responsivity of patients with IBS - a systematic review. J Psychosom Res 2022;153:110694.
16. Karling P, Nyhlin H, Wiklund U, et al. Spectral analysis of heart rate variability in patients with irritable bowel syndrome. Scand J Gastroenterol 1998;33:572–576.
17. Tanaka T, Manabe N, Hata J, et al. Characterization of autonomic dysfunction in patients with irritable bowel syndrome using fingertip blood flow. Neurogastroenterol Motil 2008;20:498–504.
18. Loria AS, Brands MW, Pollock DM, Pollock JS. Early life stress sensitizes the renal and systemic sympathetic system in rats. Am J Physiol Renal Physiol 2013;305:F390–F395.

19. Loria AS, Osborn JL. Maternal separation diminishes alpha-adrenergic receptor density and function in renal vasculature from male Wistar-Kyoto rats. Am J Physiol Renal Physiol 2017;313:F47–F54.
20. Fukudo S, Nomura T, Hongo M. Impact of corticotropin-releasing hormone on gastrointestinal motility and adrenocorticotropic hormone in normal controls and patients with irritable bowel syndrome. Gut 1998; 42:845–849.
21. Sagami Y, Shimada Y, Tayama J, et al. Effect of a corticotropin releasing hormone receptor antagonist on colonic sensory and motor function in patients with irritable bowel syndrome. Gut 2004;53:958–964.
22. Chang L, Sundaresh S, Elliott J, et al. Dysregulation of the hypothalamic-pituitary-adrenal (HPA) axis in irritable bowel syndrome. Neurogastroenterol Motil 2009; 21:149–159.
23. Udit S, Blake K, Chiu IM. Somatosensory and autonomic neuronal regulation of the immune response. Nat Rev Neurosci 2022;23:157–171.
24. Lorton D, Bellinger DL. Molecular mechanisms underlying beta-adrenergic receptor-mediated cross-talk between sympathetic neurons and immune cells. Int J Mol Sci 2015;16:5635–5665.
25. Marino F, Cosentino M. Adrenergic modulation of immune cells: an update. Amino Acids 2013;45:55–71.
26. Alaniz RC, Thomas SA, Perez-Melgosa M, et al. Dopamine beta-hydroxylase deficiency impairs cellular immunity. Proc Natl Acad Sci U S A 1999;96:2274–2278.
27. Nevin JT, Moussa M, Corwin WL, et al. Sympathetic nervous tone limits the development of myeloid-derived suppressor cells. Sci Immunol 2020;5:eaay9368.
28. Duan S, Kondo T, Miwa H, et al. Eosinophil-associated microinflammation in the gastroduodenal tract contributes to gastric hypersensitivity in a rat model of early-life adversity. Am J Physiol Gastrointest Liver Physiol 2021; 320:G206–G216.
29. Salvo-Romero E, Rodino-Janeiro BK, Albert-Bayo M, et al. Eosinophils in the gastrointestinal tract: key contributors to neuro-immune crosstalk and potential implications in disorders of brain-gut interaction. Cells 2022;11:1644.
30. Chan KY, Jang MJ, Yoo BB, et al. Engineered AAVs for efficient noninvasive gene delivery to the central and peripheral nervous systems. Nat Neurosci 2017; 20:1172–1179.
31. Dombrowicz D, Quatannens B, Papin JP, et al. Expression of a functional Fc epsilon RI on rat eosinophils and macrophages. J Immunol 2000;165:1266–1271.
32. Harima Y, Tsurutani M, Yamada S, et al. Parallel labeled-line organization of sympathetic outflow for selective organ regulation in mice. Nat Commun 2024;15:10478.
33. Powell N, Walker MM, Talley NJ. Gastrointestinal eosinophils in health, disease and functional disorders. Nat Rev Gastroenterol Hepatol 2010;7:146–156.
34. Ahrens R, Waddell A, Seidu L, et al. Intestinal macrophage/epithelial cell-derived CCL11/eotaxin-1 mediates eosinophil recruitment and function in pediatric ulcerative colitis. J Immunol 2008;181:7390–7399.
35. Matthews AN, Friend DS, Zimmerrmann N, et al. Eotaxin is required for the baseline level of tissue eosinophils. Proc Natl Acad Sci U S A 1998;95:6273–6278.
36. Thomson CA, Nibbs RJ, McCoy KD, Mowat AM. Immunological roles of intestinal mesenchymal cells. Immunology 2020;160:313–324.
37. Buechler MB, Pradhan RN, Krishnamurty AT, et al. Cross-tissue organization of the fibroblast lineage. Nature 2021;593:575–579.
38. Casado-Bedmar M, de-Faria FM, Biskou O, et al. Elevated F-EDN correlates with mucosal eosinophil degranulation in patients with IBS-A possible association with microbiota? J Leukoc Biol 2022;111:655–665.
39. Dunlop SP, Jenkins D, Spiller RC. Distinctive clinical, psychological, and histological features of postinfective irritable bowel syndrome. Am J Gastroenterol 2003; 98:1578–1583.
40. El-Salhy M, Wendelbo IH, Gundersen D. Reduced chromogranin A cell density in the ileum of patients with irritable bowel syndrome. Mol Med Rep 2013;7:1241–1244.
41. Vicario M, Gonzalez-Castro AM, Martinez C, et al. Increased humoral immunity in the jejunum of diarrhoea-predominant irritable bowel syndrome associated with clinical manifestations. Gut 2015;64:1379–1388.
42. Reber LL, Sibilano R, Mukai K, Galli SJ. Potential effector and immunoregulatory functions of mast cells in mucosal immunity. Mucosal Immunol 2015;8:444–463.
43. Walker MM, Warwick A, Ung C, Talley NJ. The role of eosinophils and mast cells in intestinal functional disease. Curr Gastroenterol Rep 2011;13:323–330.
44. Teixeira AL, Gama CS, Rocha NP, Teixeira MM. Revisiting the role of eotaxin-1/CCL11 in psychiatric disorders. Front Psychiatry 2018;9:241.
45. Friedrich M, Pohin M, Jackson MA, et al, Oxford IBD Cohort Investigators; Roche Fibroblast Network Consortium. IL-1-driven stromal-neutrophil interactions define a subset of patients with inflammatory bowel disease that does not respond to therapies. Nat Med 2021;27:1970–1981.
46. Wei K, Nguyen HN, Brenner MB. Fibroblast pathology in inflammatory diseases. J Clin Invest 2021;131:e149538.
47. De Winter BY, Deiteren A, De Man JG. Novel nervous system mechanisms in visceral pain. Neurogastroenterol Motil 2016;28:309–315.
48. Xie Z, Feng J, Hibberd TJ, et al. Piezo2 channels expressed by colon-innervating TRPV1-lineage neurons mediate visceral mechanical hypersensitivity. Neuron 2023;111:526–538.e4.
49. Nakashima C, Ishida Y, Kitoh A, et al. Interaction of peripheral nerves and mast cells, eosinophils, and basophils in the development of pruritus. Exp Dermatol 2019;28:1405–1411.
50. Pastras P, Aggeletopoulou I, Triantos C. Impact of enteric nervous cells on irritable bowel syndrome: potential treatment options. Microorganisms 2024;12:2036.
51. Leven P, Schneider R, Schneider L, et al. beta-adrenergic signaling triggers enteric glial reactivity and acute enteric gliosis during surgery. J Neuroinflammation 2023;20:255.

52. Muller PA, Schneeberger M, Matheis F, et al. Microbiota modulate sympathetic neurons via a gut-brain circuit. Nature 2020;583:441–446.
53. Matsushita N, Kobayashi K, Miyazaki J, Kobayashi K. Fate of transient catecholaminergic cell types revealed by site-specific recombination in transgenic mice. J Neurosci Res 2004;78:7–15.
54. Prabhakar S, Lule S, da Hora CC, et al. AAV9 transduction mediated by systemic delivery of vector via retro-orbital injection in newborn, neonatal and juvenile mice. Exp Anim 2021;70:450–458.

Received February 13, 2025. Accepted September 30, 2025.

Correspondence
Address correspondence to: Yi Dai, PhD, Department of Anatomy and Neuroscience, School of Medicine, Hyogo Medical University, Nishinomiya, Japan. e-mail: ydai@hyo-med.ac.jp; tel: 0798-45-6415.

Acknowledgments
The authors thank Professor Kazuto Kobayashi for providing DBH-Cre mice, and Seiko Oki, Serika Yamada, Mitsue Hagihara, and Mayumi Yamada for providing technical support during this study and preparation of the manuscript.

CRediT Authorship Contributions
Shaoqi Duan, MD, PhD (Data curation: Lead; Formal analysis: Lead; Investigation: Lead; Methodology: Lead; Writing – original draft: Lead)
Hirosato Kanda, PhD (Conceptualization: Equal; Supervision: Equal; Writing – review & editing: Equal)
Feng Zhu, PhD (Data curation: Supporting)
Masamichi Okubo, PhD (Data curation: Supporting; Writing – review & editing: Supporting)
Taro Koike, PhD (Resources: Supporting; Writing – review & editing: Supporting)
Yoshiya Ohno, PhD (Methodology: Equal; Writing – review & editing: Supporting)
Toshiyuki Tanaka, PhD (Methodology: Supporting; Writing – review & editing: Supporting)
Yukiko Harima, PhD (Methodology: Supporting)
Kazunari Miyamichi, PhD (Methodology: Supporting)
Hirokazu Fukui, MD, PhD (Resources: Supporting)
Shinichiro Shinzaki, MD, PhD (Resources: Supporting)
Yilong Cui, PhD (Writing – review & editing: Supporting)
Koichi Noguchi, MD, PhD (Conceptualization: Supporting; Supervision: Supporting; Writing – review & editing: Supporting)
Yi Dai, MD, PhD (Conceptualization: Lead; Project administration: Lead; Resources: Lead; Supervision: Lead; Writing – review & editing: Supporting)

Conflicts of interest
The authors disclose no conflicts.

Funding
This work was funded by grants from Hyogo Innovative Challenge, the Japan Society for the Promotion of Science Grants-in-Aid for Scientific Research (KAKENHI; grant numbers: 24K10501), and the Japan Society for the Promotion of Science Grant-in-Aid for Early-Career Scientists (KAKENHI; grant numbers: 22K15656)

Data Availability
Data are available upon reasonable request.

Cellular and Molecular Gastroenterology and Hepatology, Volume *20*

Supplemental information

Sympathetic Overactivation Drives Colonic Eosinophil Infiltration Linked to Visceral Hypersensitivity in Irritable Bowel Syndrome

Shaoqi Duan, Hirosato Kanda, Feng Zhu, Masamichi Okubo, Taro Koike, Yoshiya Ohno, Toshiyuki Tanaka, Yukiko Harima, Kazunari Miyamichi, Hirokazu Fukui, Shinichiro Shinzaki, Yilong Cui, Koichi Noguchi, and Yi Dai

Supplemental Table 1: Primary antibodies used for immunohistochemical staining

Antigen	Host	Manufacture	Cat. number	Dilution
Cre Recombinase	Mouse	Sigma-Aldrich	MAB3120	1:500
RFP	Rabbit	MBL	PM005	1:1000
eotaxin-1	Mouse	Santa Cruz	Sc-373767	1:50
MBP	Mouse	Bio-Rad	MCA5751	1:500
tryptase	Mouse	Abcam	ab2378	1:500
MPO	Rabbit	Abcam	Ab9535	1:1000
Iba1	Rabbit	Wako	019-1074	1:1000
CD68	Rabbit	Abcam	Ab2512	1:1000
CD31	Mouse	Millipore	MAB1393	1:1000
vimentin	Rabbit	Abcam	Ab92547	1:5000

Supplemental Table 2: Secondary antibodies used for immunohistochemical staining

Antibody	Manufacture	Cat. number	Dilution
Biotinylated-horse anti- mouse IgG	Vector	BA2001	1:200
Biotinylated-goat anti-rabbit IgG	Vector	BA1000	1:200
Donkey anti-rabbit Alexa Fluor 594	Molecular Probes	A32754	1:1000
Goat anti-rabbit Alexa Fluor 594	Molecular Probes	A11037	1:1000
Goat anti-mouse Alexa Fluor 488	Molecular Probes	A11029	1:1000
Donkey anti-goat Alexa Fluor 488	Molecular Probes	A11055	1:1000

Supplemental Table 3: Sequences of primers used in PCR

Gene	Accession No.	Type	Sequence (5′–3′)
hccl11	NM_002986.3	sense	AAGCTCACACCTTCAGCCTC
		antisense	CACTCAGGCTCTGGTTTGGT
rccl11	NM_019205.2	sense	CAGCTCTCCACAGCACTTCT
		antisense	GGGTGCCGATATTCTCCCAT
rccl24	NM_001013045.1	sense	CTTGCACCCCAGCTTTGAAC
		antisense	GGTGCTATTGCCTCGGAGTT
rccl26	XM_006249188.4	sense	GGTTCTTGAGCGTCCACACA
		antisense	TGGCTGGACACAGTATTGCT
rIL5	NM_021834.1	sense	GATGCTTCTGTGCTTGAGCG
		antisense	TCTTCGCCACACTGCTCTTT
rIL33	NM_001014166.1	sense	CTGCACAATCAGGAGACGGT
		antisense	CCCAGAAGGCACAGACCTTT
rPrg3	XM_031373412.1	sense	GCAGGGGAGAGAGTTGGTTC
		antisense	GACGCCTCCAATCCAGACAA
rTpsab1	NM_019322.2	sense	TGACTTCTACATCGCCCAGG
		antisense	Prg-GGCAGGAGTCATGTCCTTCA
rGAPDH	M17701	sense	CCAGGGCTGCCTTCTCTTGT
		antisense	CCAGCCTTCTCCATGGTGGT
mAdrb1	NM_007419.3	sense	TCTGGTCATGGGATTGCTGG
		antisense	CCTGTTGGTGACGAAATCGC
mAdrb2	NM_007420.3	sense	GAACGACAGCGACTTCTTGC

		antisense	GATCCACTGCAATCACGCAC
mAdrb3	NM_013462.3	sense	GAGTGAGTCCCCTGGAACCT
		antisense	AGTGAGGAGACAGGGATGAAA
mAdra1a	NM_001271760.1	sense	CAACCCGAGCTGCAAAGTTC
		antisense	GGAAACGTGAGCCTGAGGAA
mAdra1b	XM_011248675.4	sense	ATTGAAAGCAGACCCTCCTCG
		antisense	GCAGGTGCTGATGTGTTGTG
mAdra1d	NM_013460.5	sense	GCCACTCGCTCAAGTATCCA
		antisense	GACGATGGCTAGGGTCTTGG
mAdra2a	NM_007417.5	sense	CGCTGGACCTAGAGGAGAGT
		antisense	TTCAGCGAGCTGTTGCAGTA
mAdra2b	NM_009633.4	sense	CCAACAGTAGCGGAGCTAGG
		antisense	TGTCCAGACTGATGGCACAC
mAdra2c	NM_007418.3	sense	TCACCGTGGTAGGCAATGTG
		antisense	GCGGTAGAACGAGACGAGAG
mCD45	NM_001268286.1	sense	CCAGTGATGCTACCACAACGA
		antisense	GCACGAAGGTTGTCCAACTG
mCD31	NM_001032378.2	sense	GTGAATGACACCCAAGCGTT
		antisense	GAGCCTTCCGTTCTCTTGGT
mCD34	NM_001111059.2	sense	ACAGTACCTCACAACCCTGC
		antisense	GTCCAGGGCAAGTGCTACAT
m Pdgfra	NR_144636.1	sense	CACTTTGACCGTCCCCAAGG
		antisense	CATCCCGACCACACAAGAACA

mGAPDH	NM_001411843.1	sense	GTGTGAACGGGTGAGTTCCA
		antisense	GTCTCGCTCCTGGAAGATGG
hAdrb1	NM_000684.3	sense	ACTCGAAGCCCACAATCCTC
		antisense	TCTGGCTGGTAGTGTGTTCC
hAdrb2	NM_000024.6	sense	CTCTCATCGTCCTGGCCATC
		antisense	GAATGATCACCCGGGCCTTA
hAdrb3	NM_000025.3	sense	GCCAATTCTGCCTTCAACCC
		antisense	TCGTCAGGTTCTGGAGGGTA
hAdra1a	NM_001322502.1	sense	AATGCTTCCGACAGCTCCAA
		antisense	GATGATGCAGAGGCCCATGA
hAdra1b	NM_000679.4	sense	CTGTTGAGCTTCACCGTCCT
		antisense	ATCCTCAGGGTCAGCTCCTT
hAdra1d	NM_000678.4	sense	ACTCACTCAAGTACCCAGCC
		antisense	CTCACGGGAGAACTTGAGCA
hAdra2a	NM_000681.4	sense	GGCTACTGGTACTTCGGCAA
		antisense	CTGGTAGATGCGCACGTAGA
hAdra2b	NM_000682.7	sense	TCTGGATCGGCTACTGCAAC
		antisense	AAGGGAAGCCCAGACATTGG
hAdra2c	NM_000683.4	sense	GCCCGCTCTTCAAGTTCTTC
		antisense	CCTTGCTTGCCCATTTAGGG
hGAPDH	NM_001357943.2	sense	ACACCATGGGGAAGGTGAAG
		antisense	TGGTTCACACCCATGACGAA
